# Temporal and Context-Dependent Requirements for the Transcription Factor Foxp3 Expression in Regulatory T Cells

**DOI:** 10.21203/rs.3.rs-6596747/v1

**Published:** 2025-05-14

**Authors:** Wei Hu, Gabriel Dolsten, Giorgi Beroshvili, Eric Y. Wang, Zhong-Min Wang, Aazam P. Ghelani, Lion F. K. Uhl, Regina Bou Puerto, Xiao Huang, Anthony J. Michaels, Beatrice E. Hoyos, Wenjie Jin, Yuri Pritykin, Alexander Y. Rudensky

**Affiliations:** 1. Howard Hughes Medical Institute, Immunology Program, and Ludwig Center, Memorial Sloan Kettering Cancer Center, New York, NY 10065, USA; 2. Gerstner Sloan Kettering Graduate School of Biomedical Sciences, Memorial Sloan Kettering Cancer Center, New York, NY 10065, USA; 3. Immunology and Microbial Pathogenesis Program, Weill Cornell Graduate School of Medical Sciences, New York, NY 10021, USA; 4. Tri-Institutional MD-PhD Program, Weill Cornell Medicine, The Rockefeller University and Memorial Sloan Kettering Cancer Center, New York, NY, USA; 5. Department of Immunobiology, Yale University School of Medicine, New Haven, CT 06520, USA; 6. Institute for Biomolecular Design and Discovery, Yale University, West Haven, CT 06516, USA; 7. Computational and Systems Biology Program, Memorial Sloan Kettering Cancer Center, New York, NY, USA; 8. Lewis-Sigler Institute for Integrative Genomics and Department of Computer Science, Princeton University, Princeton, NJ, USA; 9. Present address: Immunology Discovery, Genentech Inc., South San Francisco, CA 94080, USA

## Abstract

Regulatory T (Treg) cells, expressing the transcription factor Foxp3, are obligatory gatekeepers of the immune responsiveness. While Foxp3 essential role in Treg l differentiation is well established, the mechanisms by which Foxp3 governs the Treg-specific transcriptional network remain incompletely understood. Here, we employed a novel chemogenetic system of inducible, time-controlled degradation of Foxp3 protein *in vivo* to dissect its Treg stage stage-specific functions. While Foxp3 was indispensable for the establishment of the Treg transcriptional program and suppressive function during thymic Treg progenitors and newly generated peripheral Treg cells, degradation of Foxp3 in mature Treg cells resulted in unexpectedly minimal transcriptional changes largely limited to direct Foxp3 targets and largely preserved suppressive capacity. However, tumoral Treg cells were uniquely sensitive to Foxp3 degradation, which led to impaired suppressive function and tumor growth restraint absent pronounced adverse effects. These studies demonstrate context-dependent differential requirement for Foxp3 for Treg transcriptional and functional programs.

## Introduction

Regulatory T (Treg) cells are requisite watchmen of the immune system, whose identity is distinguished by the expression of their X-chromosome encoded lineage specifying transcription factor (TF) Foxp3^1, 2, 3^. Foxp3 plays a critical role in Treg differentiation, conferring both suppressor function and fitness, largely by exploiting the pre-established epigenetic landscape in precursor cells prior to Foxp3 expression^4, 5, 6^. Genetic studies in mice on a non-autoimmune prone genetic background showed that Foxp3 expression is stable in fully differentiated Treg cells in both physiological and inflammatory settings, while recently generated Treg cells can lose Foxp3 expression^7, 8, 9^. Further, in inflammatory settings, Treg cells upregulate Foxp3 expression and increase their proliferative fitness and suppressor function^7^. Nevertheless, Treg cell functionality and Foxp3 expression can become compromised in severe infections or autoimmune disease settings, in particular in conjunction with genetic predispositions or IL-2 deprivation^10, 11^.

The indispensable role of Foxp3 in establishing identity and functionality of Treg cells during their differentiation has been demonstrated by comparisons of mice expressing a *Foxp3*^*GFPKO*^ reporter null versus a functional *Foxp3*^*GFP*^ allele^4, 12^. The absence of a functional Foxp3 allele impaired Treg cell development and led to early-onset systemic autoimmune disease, underscoring the essential role of Foxp3 in conferring Treg functionality^1, 13^. The currently prevailing notion of a requirement for continuous Foxp3 expression was suggested by a Treg loss-of-function observed upon Cre recombinase induced ablation of a *Foxp3* conditional allele *in vitro* in rapidly dividing Treg cells followed by their adoptive transfers into lymphopenic hosts^14^. However, the confounders of the design of these early studies left unresolved the question of whether the Foxp3 dependent Treg functional program is intrinsically resilient or vulnerable. This uncertainty, besides its major basic and clinical significance, is particularly intriguing in light of the recent studies suggesting a model where Foxp3 is acting largely indirectly by inducing relatively modest changes in the expression of few, yet to be defined direct target genes, which in turn can act in a ‘relay’-like manner to establish genome-wide transcriptional and functional program of Treg cells^12^. Thus, a role for Foxp3 in transcriptional and functional features of differentiated Treg cells remains unknown despite major previous efforts.

Here we sought to investigate the role of Foxp3 during Treg cell differentiation, maintenance, and turnover using a novel chemogenetic model, which enabled punctual inducible degradation of Foxp3 protein *in vivo*. By analyzing the transcriptional and functional features of Treg cells following short term Foxp3 protein degradation, we found that Foxp3 was essential for the establishment of gene expression program and suppressor function during thymic Treg cell differentiation and in recently differentiated Treg cells. Contrary to complete loss of Treg-mediated suppression resulting from developmental Foxp3 deficiency or Treg cell ablation, Foxp3-degraded mature Treg cells largely maintained their suppressive capacity, both *in vivo* and *ex vivo*. Accordingly, Foxp3 degradation led to minimal gene expression changes limited to a small group of genes enriched for direct Foxp3 targets. Interestingly, induced Foxp3 protein degradation preferentially destabilized intratumoral Treg cells, leading to a loss of suppressor function and tumor rejection. This study reveals differential roles of Foxp3 at different Treg developmental stages, shedding light on its unique mode of action as a transcription factor.

## Results

### Inducible Foxp3 protein degradation *in vivo*

Despite being central to Treg biology, the role Foxp3 protein expression in developing vs. mature Treg cells – particularly the mechanisms underlying the vulnerabilities and resilience of Foxp3-dependent gene regulatory network in early-life vs. adulthood, as well as in health vs. disease - remains unknown. A major obstacle to gaining this insight has been the limitations of *Foxp3* gene ablation strategies for dissecting gene regulatory programs, as the prolonged turnover of Foxp3 RNA and protein following gene deletion confounds the distinction between direct and indirect effects.

**Extended Data Figure 1.**
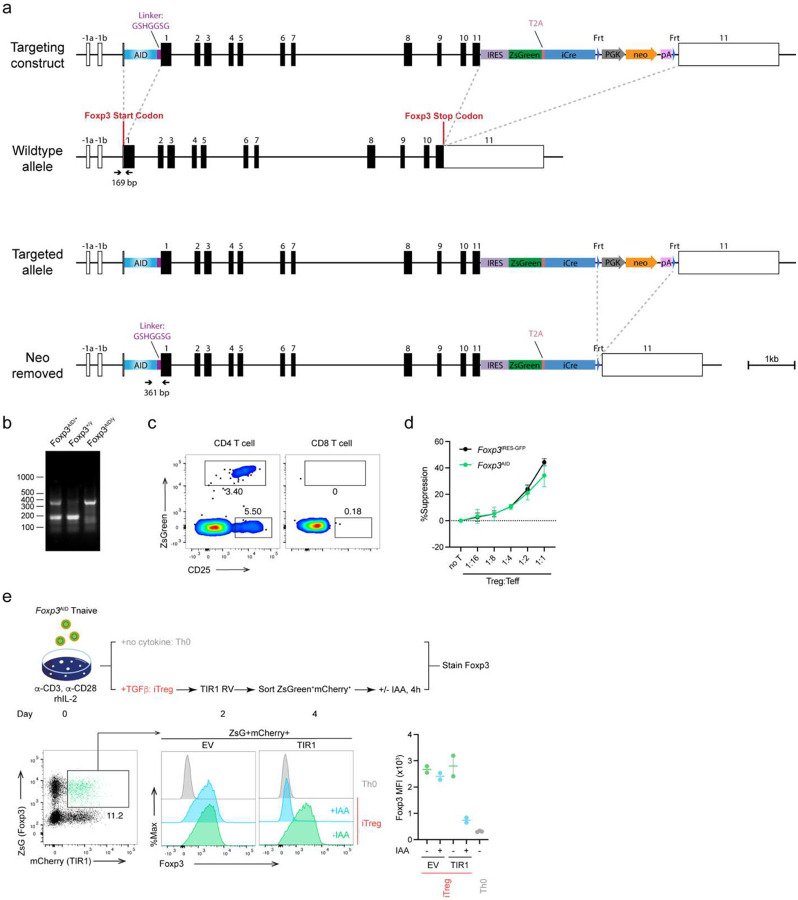
Generation of *Foxp3*^AID^ mice. **(a)** Gene targeting strategy. The auxin-inducible degron (AID) sequence was fused to the N-terminus of Foxp3 via a seven-amino acid flexible linker. An IRES-ZsGreen-T2A-iCre-Frt-neo-Frt cassette was inserted into the 3′ UTR. Arrows indicate the locations of PCR primers used to distinguish *Foxp3*^WT^ and *Foxp3*^AID^ alleles. IRES: internal ribosome entry sequence. iCre: codon improved Cre recombinase. PGK: PGK promoter. Neo: neomycin resistant gene. pA: bGH polyA sequence. **(b)** PCR validation of the knock-in allele. **(c)** Expression pattern of the ZsGreen reporter in *Foxp3*^AID/WT^ heterozygous females. ZsGreen expression was restricted to CD25^+^ CD4 T cells, consistent with Foxp3 expression. **(d)** ZsGreen^+^ *Foxp3*^AID^ Treg cells suppressed naïve CD4^+^ T cell proliferation comparably to *Foxp3*^GFP^ Treg cells *in vitro*. Line graph represents mean ±SEM of two biological replicates. **(e)** Naïve CD4^+^ T cells from *Foxp3*^AID^ mice were cultured under Treg inducing conditions and transduced with either a TIR1-encoding retrovirus or the empty vector control. AID-tagged Foxp3 protein was selectively degraded in TIR1-transduced induced Treg (iTreg) cells upon indole-acetic acid (IAA) treatment. Scatter plots represents mean ±SEM.

**Extended Data Figure 2.**
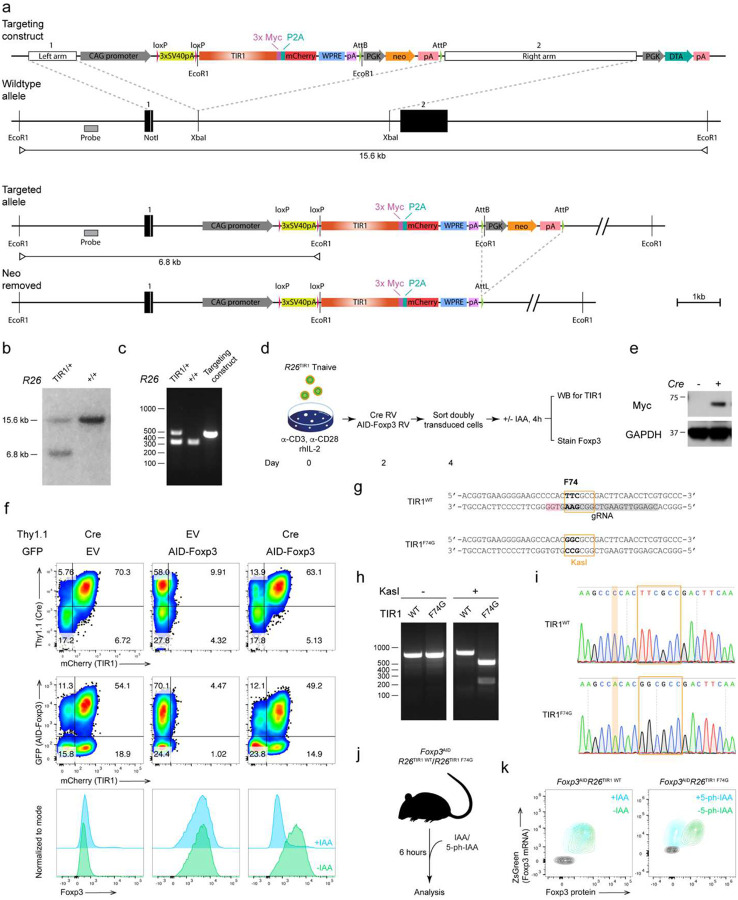
Generation of *ROSA26*^TIR1^ and *ROSA26*^TIR1(F74G)^ mice. **(a**) Gene targeting strategy. WPRE: Woodchuck hepatitis virus post-transcriptional regulatory element; DTA: Diphtheria toxin fragment A). **(b)** Southern blot validation of heterozygous *ROSA26*^TIR1/+^ mice using the hybridization probe shown in (a). **(c)** PCR validation of *ROSA26*^TIR1/+^ mice. **(d–f)** Naïve CD4^+^ T cells from *ROSA26*^TIR1/+^ mice were co-transduced with retroviruses expressing Cre and AID-Foxp3 (d). TIR1 expression was induced in a Cre-dependent manner (e), resulting in AID-Foxp3 degradation upon IAA treatment (f). **(g)** Guide RNA (gRNA) design for CRISPR-mediated F74-to-G mutation in TIR1. The gRNA seed sequence is shown in grey; the PAM sequence is in pink. The F74G mutation creates a KasI restriction site. **(h–i)** Validation of the F74G mutation by KasI digestion (h) and Sanger sequencing (i). **(j–k)** The TIR1 F74G mutation enables *in vivo* protein degradation in response to 5-ph-IAA.

**Figure 1.**
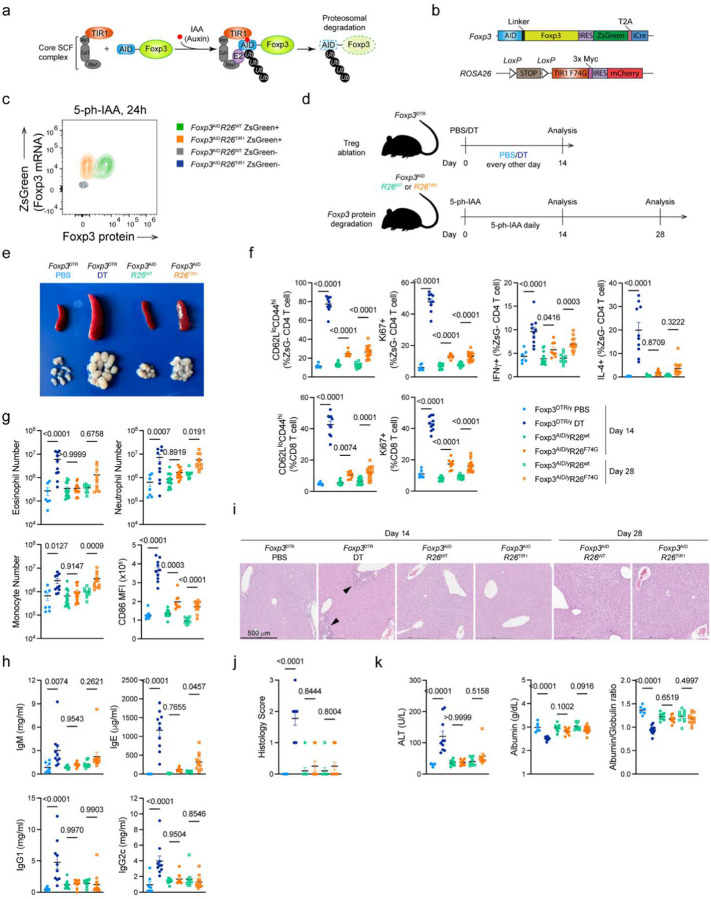
Foxp3 degradation causes minimal immune activation in adult lymphoreplete mice. **(a)** Schematic of the inducible Foxp3 protein degradation model. **(b)** Schematic of the *Foxp3*^AID^ and *R26*^TIR^ alleles. **(c)** Flow cytometry plot showing 5-ph-IAA induced Foxp3 protein degradation. **(d)** Experimental design. **(e)** Size of spleen and lymph nodes after 14 days of Treg ablation or Foxp3 degradation. **(f)** Activation, proliferation, and cytokine production of CD4 (upper) and CD8 (lower) T cells following Treg ablation or Foxp3 degradation. **(g)** Number of eosinophils, neutrophils, monocytes, and CD86 levels on dendritic cells following Treg ablation or Foxp3 degradation. **(h)** Serum antibody levels following Treg ablation or Foxp3 degradation. **(i-j)** Representative H&E stain (i) and histology scores (j) of the liver following Treg ablation or Foxp3 degradation. **(k)** Liver damage measured by serum ALT, albumin, and albumin/globulin ratio. Scatter plots represent mean ± SEM. Data are pooled from two independent experiments. One-way ANOVA.

To overcome these limitations, we generated a new chemogenetic mouse model that enables rapid drug-inducible Foxp3 protein degradation *in vivo* (Figure 1a–b). In this model, based on the auxin-sensing pathway in plants^15^, the endogenous *Foxp3* allele encoding auxin-inducible degron (AID)-Foxp3 fusion protein alongside a zsGreen transcriptional reporter and Cre recombinase (Extended Data Figure 1 a–b) was combined with the *ROSA26* (*R26*) allele harboring a plant derived E3 ligase TIR1 and mCherry reporter preceded by a loxP-flanked STOP cassette (Extended Data Figure 2a–c). The resulting *Foxp3*^AID^ mice exhibited the expected Foxp3 expression pattern (Extended Data Figure 1c) limited to ZsGreen^+^ Treg cells, whose suppressive capacity was similar to that of *Foxp3*^GFP^ Treg cells^16^ (Extended Data Figure 1d). Upon the addition of indole acetic acid (IAA) to TIR1-expressing *Foxp3*^AID^ Treg cells *in vitro*, AID-fused Foxp3 underwent poly-ubiquitination and proteasomal degradation in a TIR1 dependent manner^17^ (Extended Data Figure 1e, Extended Data Figure 2d–f). Since we found the *in vivo* performance of the original AID-Foxp3-TIR1 protein degradation system suboptimal (Extended Data Figure 2j–k), we mutated TIR1 phenylalanine 74 to a glycine in *R26*^TIR1^ mice using CRISPR mediated gene editing (Extended Data Figure 2g–k). Instead of unmodified IAA, the mutant TIR1(F74G) protein recognizes 5-phenyl-IAA (5-ph-IAA)^18^. This improved degradation in *Foxp3*^AID^*R26*^TIR1(F74G)^ mice enabled rapid and near complete *in vivo* degradation of the Foxp3 protein within 6 hours upon 5-ph-IAA administration (Extended Data Figure 2j–k). The effect persisted for at least 24 hours post drug administration (Figure 1c), ensuring continuous Foxp3 degradation upon once daily 5-ph-IAA administration.

### Foxp3 expression is largely dispensable for preventing autoimmune inflammatory disease cells in adulthood

**Extended Data Figure 3.**
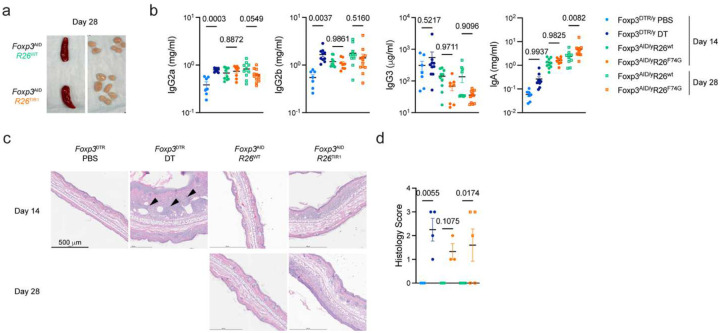
Foxp3 protein degradation in adult lymphoreplete mice induces minimal immune activation. **(a)** Size of the spleen and lymph nodes after 28 days of Foxp3 degradation. **(b)** Serum antibody levels following Treg ablation or Foxp3 degradation. **(c-d)** Representative H&E stain (c) and histology scores (d) of the skin following Treg ablation or Foxp3 degradation. Data are pooled from two independent experiments. Scatter blots represent mean ±SEM. One-way ANOVA.

**Extended Data Figure 4.**
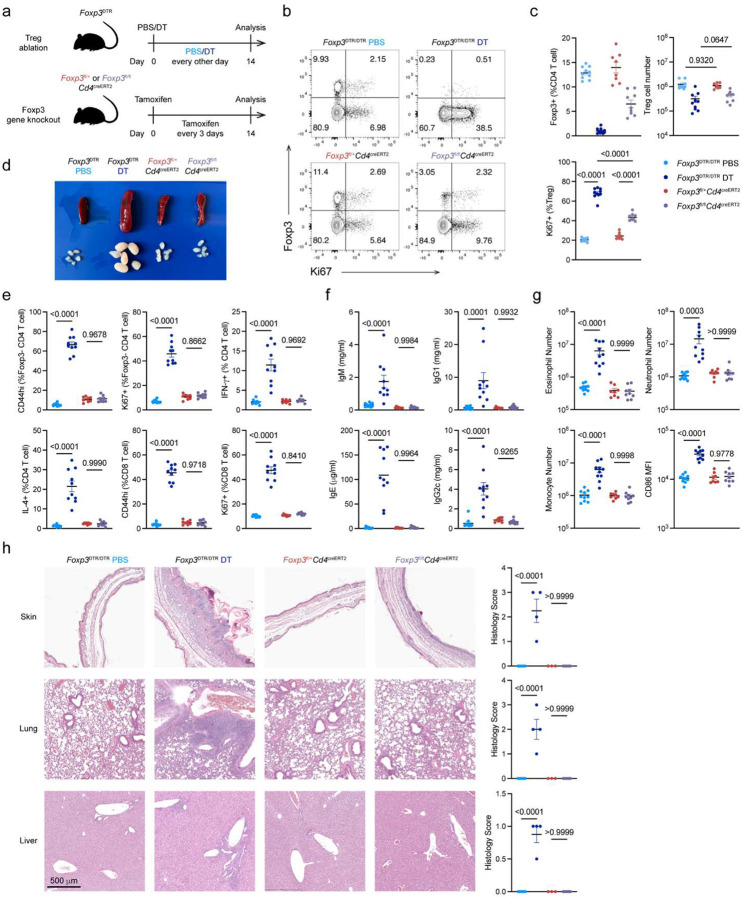
Inducible Foxp3 gene knockout causes minimal immune activation in adult lymphoreplete mice. **(a)** Experimental design. **(b-c)** Representative plot (b) and combined data (c) showing the efficiency of Foxp3 gene knockout. **(d)** Size of the spleen and lymph nodes after 14 days of inducible Foxp3 gene knockout. **(e-g)** T cell activation (e), serum antibody levels (f), and myeloid cell expansion (g) following 14 days of inducible Foxp3 gene knockout. **(h)** Representative H&E stain (left) and histology scores (right) of the skin, liver, and lung following 14 days of Treg ablation or Foxp3 degradation. Data are pooled from two independent experiments. Scatter blots represent mean ±SEM. One-way ANOVA.

Using the continuous *in vivo* degradation of the Foxp3 protein, we investigated its role in Treg maintenance and function in adult mice using a side-by-side analysis of *Foxp3*^AID^*R26*^TIR1(F74G)^ treated with 5-ph-IAA and *Foxp3*^DTR^ mice subjected to Treg cell ablation upon administration of diphtheria toxin (DT) (Figure 1d). While the continuous Treg depletion led to flagrant splenomegaly and lymphadenopathy, these manifestations were unexpectedly mild following continuous Foxp3 degradation in *Foxp3*^AID^*R26*^TIR1(F74G)^ mice treated with 5-ph-IAA for the same duration (Figure. 1e). Accordingly, DT-mediated Treg ablation induced pronounced T, B and myeloid cell activation, whereas Foxp3 degradation only had minimal effects (Figure 1f–h). Most Treg cell depleted *Foxp3*^DTR^ mice succumb to the resulting autoimmune syndrome within two-three weeks^19^. In sharp contrast, continuous Foxp3 degradation for four weeks did not result in any noticeable clinical manifestations of autoimmune disease with only mildly increased state of immune cell activation observed (Figure. 1f–h, Extended Data Figure 3a–b). In this regard, hepatitis and liver damage, associated with a marked immune infiltration, elevated serum alanine aminotransferase (ALT) and diminished albumin in Treg-depleted *Foxp3*^DTR^ mice, were undetectable in *Foxp3*^AID^*R26*^TIR1(F74G)^ mice after four weeks of continuous Foxp3 degradation (Figure 1i–k). Notably, immune cell activation and tissue inflammation following Foxp3 degradation appeared to reach a new setpoint, as no further increases were observed between two and four weeks of 5-ph-IAA treatment (Figure 1f–k, Extended Data Figure 3b–d). These data suggest that contrary to an absolute requirement for Foxp3^+^ Treg cells for the restraint of fatal autoimmune inflammation under physiological conditions, Foxp3 protein in differentiated Treg cells was largely dispensable for their suppressor function. The preserved Treg functionality was unlikely due to the minimal residual Foxp3 protein as Treg cells from adult *Cd4*^creERT2^*Foxp3*^fl^ mice retained their function even after tamoxifen-induced *Foxp3* gene ablation (Extended Data Figure 4).

### Foxp3 degradation impacts expression of a small group of genes in mature Treg cells

**Extended Data Figure 5.**
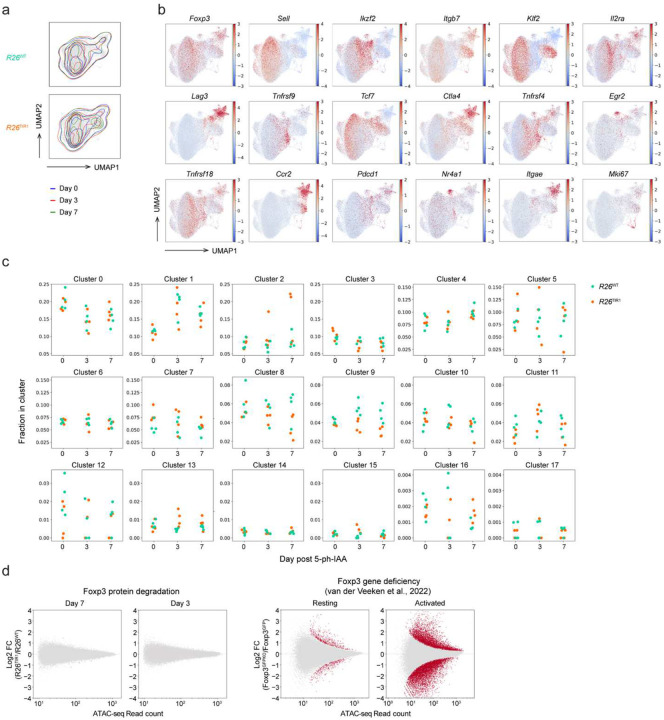
Foxp3 degradation in developed Treg cells induces minimal gene expression and chromatin accessibility changes. (a) UMAP visualization of scRNA-seq data from *Foxp3*^AID^*R26*^WT^ and *Foxp3*^AID^*R26*^TIR1(F74G)^ Treg cells on days 0, 3 and 7 of 5-ph-IAA–induced Foxp3 degradation. (b) UMAP visualization of representative genes from the single cell RNA-seq dataset colored by expression level. **(c)** Scatter plot showing the fraction of *Foxp3*^AID^*R26*^WT^ and *Foxp3*^AID^*R26*^TIR1(F74G)^ Treg cells in each cluster defined in Figure 2c. Data is summarized in Figure 2d. **(d)** MA plot showing differentially accessible ATAC-seq peaks induced by Foxp3 degradation (left) and Foxp3 gene deficiency (right).

**Figure 2.**
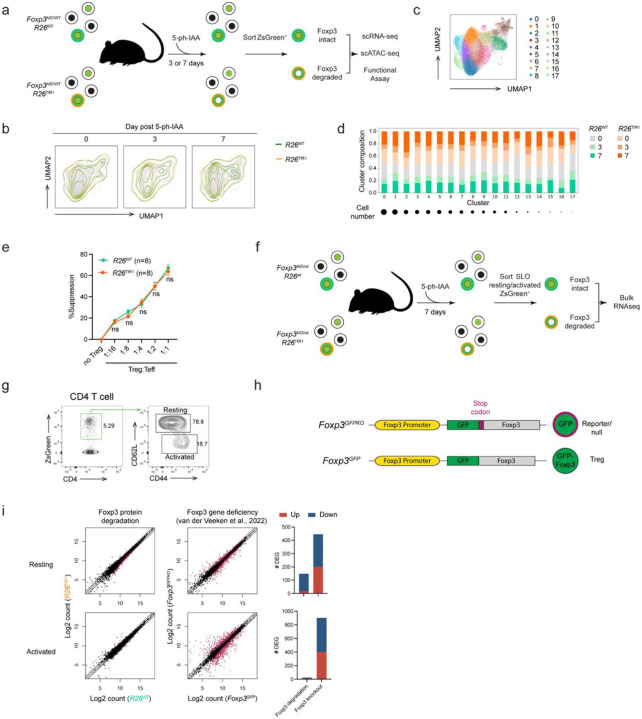
Foxp3 degradation induces minimal gene expression and functional changes in mature Treg cells. **(a)** Experimental design of single-cell RNA-seq, ATAC-seq, and functional assays. Each genotype and time point consisted of four independent biological replicates. **(b)** UMAP visualization of scRNA-seq data from *Foxp3*^AID^*R26*^WT^ and *Foxp3*^AID^*R26*^TIR1(F74G)^ Treg cells before and 3 or 7 days after 5-ph-IAA–induced Foxp3 degradation. **(c)** UMAP visualization of the same scRNA-seq data, colored by identified clusters. **(d)** Proportions of *Foxp3*^AID^*R26*^WT^ and *Foxp3*^AID^*R26*^TIR1(F74G)^ Treg cells from each time point within each cluster. **(e)** *In vitro* suppression assay of Treg cells sorted from *Foxp3*^AID^*R26*^WT^ and *Foxp3*^AID^*R26*^TIR1(F74G)^ mice after 7 days of *in vivo* 5-ph-IAA treatment. 5-ph-IAA was included in culture to sustain Foxp3 degradation. Line graph represents mean ± SEM. Data are pooled from two independent experiments, multiple *t-*tests. **(f)** Experimental design of the bulk RNA-seq analysis. Each genotype and time point consisted of three independent biological replicates. **(g)** Gating strategy for sorting resting and activated Treg cells. **(h)** Schematic comparison of the *Foxp3*^GFPKO^ reporter null allele and the functional *Foxp3*^GFP^ allele. **(i)** Scatter plots and bar graphs showing the number of differentially expressed genes in resting or activated Treg cells caused by Foxp3 protein degradation or genetic Foxp3 deficiency.

To gain insights into the mechanisms of the observed retention of mature Treg cell identity and function upon Foxp3 protein loss, we analyzed its effect on Treg cell gene expression at a single cell resolution using RNA (scRNA-seq) and ATAC sequencing (scATAC-seq) of FACS-sorted ZsGreen^+^ cells from the secondary lymphoid organs of *Foxp3*^AID^*R26*^TIR1(F74G)^ mice and *Foxp3*^AID^*R26*^WT^ controls on day 3 and 7 of continuous 5-ph-IAA treatment (Figure 2a). The mildly increased inflammation observed in hemizygous *Foxp3*^AID^ males following Foxp3 degradation may influence gene transcription through cell-extrinsic mechanisms, potentially confounding cell-intrinsic gene expression changes. To avoid these effects, we performed the experiment in *Foxp3*^AID/WT^ heterozygous females. Because the X-linked Foxp3 gene undergoes random X chromosome inactivation, *Foxp3*^AID/WT^ females harbor a mixed population of Treg cells expressing either the *Foxp3*^WT^ or *Foxp3*^AID^ allele. Upon 5-ph-IAA treatment, the *Foxp3*^WT^-expressing Treg cells remain unaffected and maintain comparable immune tone in experimental and control groups of mice, thereby isolating the effects of Foxp3 degradation on *Foxp3*^AID^-expressing Treg cells. Consistent with minimal immune activation following Foxp3 degradation, our analysis of global zsGreen^+^ cell transcriptomes, visualized by uniform manifold approximation and projection (UMAP), revealed minute differential gene expression on both day 3 and day 7 induced upon Foxp3 degradation (Figure 2b). Similarly, overlaying the UMAP plots of *Foxp3*^AID^*R26*^TIR1(F74G)^ Treg cells on day 0, 3 and 7 of 5-ph-IAA treatment revealed minor changes (Extended Data Figure 5a). To account for potential transcriptional changes within rare Treg subpopulations, we performed Leiden clustering yielding 18 cell clusters (Figure 2c; Extended Data Figure 5b). *Foxp3*^AID^*R26*^WT^ and *Foxp3*^AID^*R26*^TIR1(F74G)^ Treg cells from all three time points were similarly represented in most of these clusters indicative of minimal transcriptional changes across the entire Treg cell population (Figure 2d, Extended Data Figure 5c). Consistent with these findings, 5-ph-IAA induced Foxp3 degradation in sorted *Foxp3*^AID^*R26*^TIR1(F74G)^ Treg cells did not impact their ability to suppress CD4 T cell proliferation *in vitro* when compared to similarly treated *Foxp3*^AID^*R26*^WT^ Treg cells (Figure 2e).

Because of its known limitation in capture efficiency, we complemented scRNA-seq analysis with bulk gene expression analysis of resting and activated ZsGreen^+^ cells sorted from *Foxp3*^AID^*R26*^TIR1(F74G)^ and *Foxp3*^AID^*R26*^WT^ mice on day 7 of 5-ph-IAA treatment, the time point of more pronounced effects of Foxp3 degradation observed at a single cell level (Figure 2f–g). Like the scRNA seq experiment, bulk RNA-seq revealed only a very small group of genes differentially expressed in both resting and activated Treg cells upon Foxp3 protein degradation (Figure 2i). In contrast, both activated and resting cells expressing a *Foxp3*^GFPKO^ reporter-null allele^4^, which have never expressed Foxp3 protein, exhibited markedly more pronounced differential gene expression in comparison to Foxp3-sufficient *Foxp3*^GFP^ Treg cells^12^ (Figure 2h–i). Likewise, scATAC-seq analysis of Foxp3-degraded resting and activated *Foxp3*^AID^ Treg cells revealed minimal changes in chromatin accessibility, unlike the substantial differences observed between reporter-null *Foxp3*^GFPKO^ Treg cells and Foxp3-sufficient *Foxp3*^GFP^ Treg cells in both resting and activated states^12^ (Extended Data Figure 5d).

**Extended Data Figure 6.**
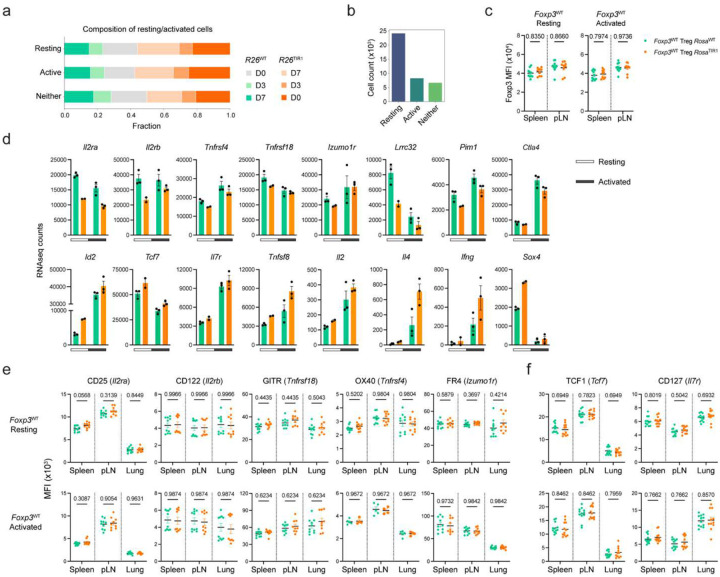
Foxp3 degradation in developed Treg cells altered the expression of a small group of genes. **(a)** The composition of *Foxp3*^AID^*R26*^WT^ and *Foxp3*^AID^*R26*^WT(F74G)^ Treg cells within resting and activated subsets from day 0, day 3, and day 7 of Foxp3 degradation. **(b)** Number of resting and activated Treg cells utilized for the differential gene expression analysis in Figure 3. **(c)** Flow cytometry analysis of Foxp3 protein and mRNA levels (reported by ZsGreen) in *Foxp3*^WT^ Treg cells from heterozygous *Foxp3*^AID/WT^*R26*^WT^ and *Foxp3*^AID/WT^*R26*^TIR1(F74G)^ females after 7 days of Foxp3 degradation. Scatter plots represent mean ± SEM. Data are pooled from two independent experiments. Two-way ANOVA. **(d)** Bulk RNA-seq read counts of indicated genes in *Foxp3*^AID^ Treg cells from heterozygous *Foxp3*^AID/WT^*R26*^WT^ and *Foxp3*^AID/WT^*R26*^TIR1(F74G)^ females after 7 days of Foxp3 degradation. Bar graphs represent mean ± SEM. **(e)** Flow cytometry analysis of CD25, CD122, OX40, GITR, and FR4 protein levels in *Foxp3*^WT^ Treg cells from heterozygous *Foxp3*^AID/WT^*R26*^WT^ and *Foxp3*^AID/WT^*R26*^TIR1(F74G)^ females after 7 days of Foxp3 degradation. **(f)** Flow cytometry analysis of CD127 and TCF1 protein levels in *Foxp3*^AID^ Treg cells from heterozygous *Foxp3*^AID/WT^*R26*^WT^ and *Foxp3*^AID/WT^*R26*^TIR1(F74G)^ females after 7 days of Foxp3 degradation. (e-f) Scatter plots represent mean ± SEM. Data are pooled from two independent experiments. Multiple *t*-tests.

**Figure 3.**
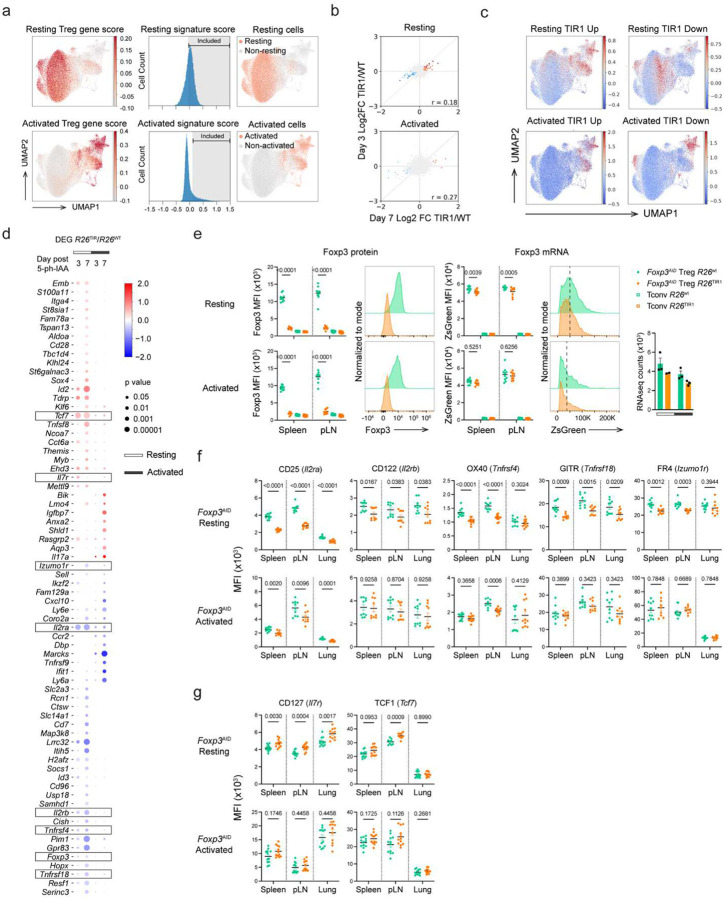
Foxp3 degradation in mature Treg cells induces expression changes in a limited set of genes. **(a)** Treg cells from the scRNA-seq dataset were classified as resting or activated based on exceeding the threshold for resting or activated gene signature scores and were subsequently analyzed. **(b)** Scatter plot showing the correlation of gene expression changes induced by Foxp3 degradation at day 3 and day 7 in resting and activated Treg cells. **(c)** UMAP visualization of resting and activated Treg cells colored by gene signature scores for the “TIR1-up” and “TIR1-down” gene sets, up- and downregulated upon Foxp3 degradation, respectively. Data are pooled from two independent experiments. One-way ANOVA. **(d)** Dot plot summarizing statistically significant differentially expressed genes in resting or activated Treg cells following 3 or 7 days of 5-ph-IAA–induced Foxp3 degradation. **(e)** Flow cytometry analysis of Foxp3 protein and mRNA levels (reported by ZsGreen) in *Foxp3*^AID^ Treg cells from heterozygous *Foxp3*^AID/WT^*R26*^WT^ and *Foxp3*^AID/WT^*R26*^TIR1(F74G)^ females after 7 days of Foxp3 degradation. Scatter plots represent mean ± SEM. Data are pooled from two independent experiments. Two-way ANOVA. **(f)** Flow cytometry analysis of CD25, CD122, OX40, GITR, and FR4 protein levels in *Foxp3*^AID^ Treg cells from heterozygous *Foxp3*^AID/WT^*R26*^WT^ and *Foxp3*^AID/WT^*R26*^TIR1(F74G)^ females after 7 days of Foxp3 degradation. **(g)** Flow cytometry analysis of CD127 and TCF1 protein levels in *Foxp3*^AID^ Treg cells from heterozygous *Foxp3*^AID/WT^*R26*^WT^ and *Foxp3*^AID/WT^*R26*^TIR1(F74G)^ females after 7 days of Foxp3 degradation. (f-g) Scatter plots represent mean ± SEM. Data are pooled from two independent experiments. Multiple *t*-tests.

To gain further insights into Foxp3’s transcriptional role in mature Treg cells, we performed additional analyses on the small group of genes whose expression was affected by Foxp3 degradation. To ensure that the activation status did not confound these analyses, we defined cells in our scRNA-seq dataset as resting and activated using the gene scores based on previously identified resting and activated Treg transcriptional signatures^12^ (Figure 3a, Extended Data Figure 6a–b). We then performed pseudo-bulk differential gene expression analyses for the resting and activated Treg cells. Gene expression changes on day 3 and day 7 post Foxp3 degradation were well correlated, with a larger fold change observed on day 7 indicating time-dependent augmentation of gene expression changes (Figure 3b). We classified the latter into four groups: genes upregulated (“resting TIR1-up” and “activated TIR1-up”) and downregulated (“resting TIR1-down” and “activated TIR1-down”) upon Foxp3 degradation in resting and activated Treg cells, respectively. A closer examination of Foxp3 regulated transcripts in resting Treg cells showed that the “resting TIR1-up” genes repressed by Foxp3 were expressed more highly in activated vs. resting Treg cells, whereas “resting TIR1-down” genes induced by Foxp3 genes showed the opposite pattern (Figure 3c). This result suggests that in resting Treg cells, Foxp3 enables a ”goldilocks” state of expression of T cell activation-associated genes.

The gene set with statistically significant differential expression caused by Foxp3 degradation in either resting or activated Treg cells included 32 TIR1-up and 38 TIR1-down genes repressed and activated by Foxp3, respectively (Figure 3d). The latter group included *Foxp3* gene itself. Indeed, ZsGreen expression, reporting *Foxp3* mRNA levels, showed a slight but statistically significant reduction in resting Treg cells at day 7, consistent with the bulk RNA-seq analysis (Figure 3e, Extended Data Figure 6c). Flow cytometric analyses also showed Foxp3 degradation-induced reduction in CD25 (*Il2ra*) expression in both resting and activated Treg cells (Figure 3f; Extended Data Figure 6d–e), whereas reduction in CD122 (*Il2rb*), OX40 (*Tnfrsf4*), GITR (*Tnfrsf18*) and FR4 (*Izumo1r*) levels was limited to resting Treg cells (Figure 3f; Extended Data Figure 6d–e). On the flip side, CD127 (*Il7r*) and TCF1 (*Tcf7*) protein expression was increased in resting Treg cells following Foxp3 degradation, consistent with the observed changes in their transcript levels (Figure 3g, Extended Data Figure 6d,f).

### Foxp3 degradation sensitive genes are enriched for Foxp3 binding

**Extended Data Figure 7.**
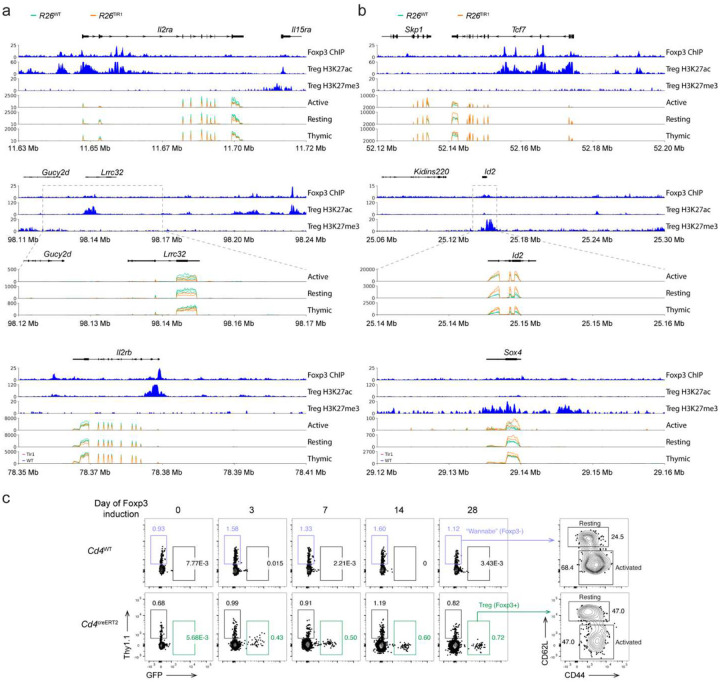
Genes sensitive to Foxp3 degradation in mature Tregs are potentially enriched for direct Foxp3 targets. **(a–b)** Representative tracks showing Foxp3 ChIP, Treg H3K27Ac, Treg H3K27me3, and RNA-seq profiles in activated, resting, and nascent thymic Treg cells for candidate Foxp3-activated (a) and Foxp3-repressed (b) genes. **(c)** Representative flow cytometry plots and gating strategy for RNA-seq analysis of Treg “wannabe” cells with or without Foxp3 induction at the indicated time points following 4-OHT administration.

**Figure 4.**
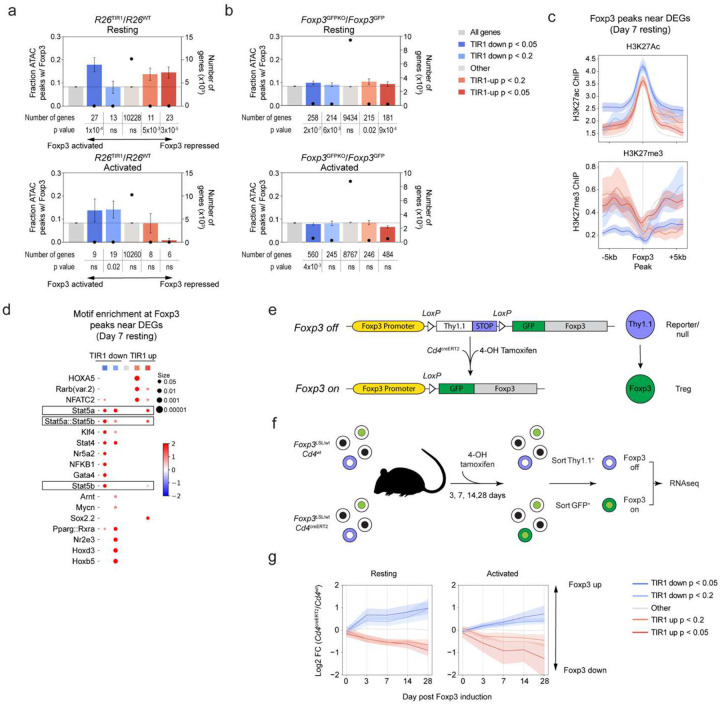
Foxp3 degradation-sensitive genes in mature Treg cells are enriched for Foxp3 binding. **(a)** Bar graphs showing the proportion of ATAC-seq peaks near Foxp3 degradation-induced differentially expressed genes (DEGs) bound by Foxp3. Genes are stratified by statistical significance (p-values) in resting and activated Treg cells. **(b)** Bar graphs showing the proportion of ATAC-seq peaks near Foxp3-dependent bound by Foxp3. Genes are stratified by p-values in resting and activated Treg cells. **(c)** H3K27Ac and H3K27me3 ChIP-seq signals at Foxp3-bound ATAC-seq peaks near Foxp3 degradation–induced DEGs. **(d)** Dot plot showing transcription factor motif enrichment within Foxp3-bound regions near Foxp3 degradation–induced DEGs. **(e)** Schematic diagram illustrating the “on” and “off” states of the reversible reporter-null *Foxp3*^LSL^ allele. **(f)** Experimental design of the gain-of-function experiment to induce Foxp3 expression in Treg “wannabe” cells. Each genotype and time point consisted of two independent biological replicates. **(g)** Line graph depicting gene expression changes across different time points following Foxp3 induction in Treg “wannabe” cells.

Previous studies of differential gene expression in resting and activated *Foxp3*^GFP^ Treg and *Foxp3*^GFPKO^ Treg “wannabe” cells identified the overall Foxp3-dependent gene set without distinguishing between potential direct and indirect Foxp3 targets^4, 5^. Given the short duration of Foxp3 degradation and the small number of genes impacted by it, we reasoned that Foxp3 degradation sensitive genes are likely enriched for direct Foxp3 transcriptional targets. To discern Foxp3-degradation sensitive from Foxp3-dependent transcriptional features, we first grouped the gene expression data from both comparisons in both resting and activated Treg cells based on the p values (Figure 4a–b). We then examined the number of Foxp3 binding sites near the stratified genes in each group. As Foxp3 is known to bind predominantly to open chromatin regions and its global genome occupancy is not associated with Foxp3-dependent gene expression or chromatin accessibility changes, we normalized the number of Foxp3 peaks to the number of open chromatin regions surrounding each gene, using Foxp3 CUT&RUN and Treg ATAC-seq datasets from previous studies^12^. Notably, the top “TIR1-down” Foxp3 degradation-sensitive genes in resting Treg cells contained significantly more Foxp3 binding sites per gene (Figure 4a). This observation suggests that genes in this group, such as *Il2ra*, *Lrrc32*, and *Il2rb* known for their role in Treg differentiation, maintenance, and functionality^20, 21^, are extensively bound and likely directly induced by Foxp3 (Extended Data Figure 7a). Likewise, the top “TIR1-up” Foxp3-degradation sensitive genes in resting Treg cells also contained more Foxp3 binding sites per gene (Figure 4a). Genes in this group, including *Tcf7*, *Id2*,and *Sox4*, have been implicated in Treg cell gene expression and optimal function^22, 23, 24, 25, 26^ (Extended Data Figure 7b). We hereby refer to these two gene groups as “Foxp3-activated” and “Foxp3 repressed” genes, respectively. Of note, when the entirety of Foxp3-dependent genes expressed in resting and activated Treg cells was stratified and analyzed in the same fashion, no enrichment for Foxp3 binding was observed, likely because a larger number of indirect Foxp3 target genes obscured small number of direct ones (Figure 4b). In contrast to resting Treg cells, similar analyses of Foxp3 degradation-sensitive genes in their activated counterparts did not reveal significant enrichment of Foxp3 binding in any gene groups, suggesting that as upon Treg activation direct gene regulation by Foxp3 can be compensated by some other T cell activation-dependent transcription factors (Figure 4a–b). This observation was also consistent with the smaller number of DEGs identified by both bulk and scRNA-seq analyses of Foxp3 degradation in activated Treg cells (Figure 2i and 3d). We next explored additional features of the “Foxp3-activated” and “Foxp3 repressed” genes besides the observed enrichment for Foxp3 binding. Compared to other gene groups not enriched for Foxp3 binding, “Foxp3-activated” genes showed the highest level of the activating H3K27Ac and the lowest level of the repressive H3K27me3 histone modifications, while “Foxp3 repressed genes showed the opposite pattern for these two histone modifications (Figure 2c). In addition, motif enrichment analysis of Foxp3-bound ATAC-seq peaks near “Foxp3-activated” genes revealed a pronounced enrichment for STAT binding motifs (Figure 4d). This finding is consistent with the inclusion of both *Il2ra* and *Il2rb* within this gene set, raising a possibility that Stat5 cooperates with Foxp3 to drive their activation.

### Long time scale of establishment of Foxp3-dependent gene

To complement Foxp3-degradation based “loss-of-function” studies, we employed a “gain-of-function” approach using a reversible *Foxp3*^loxP-Thy1.1-STOP-loxP-GFP^ reporter null allele (*Foxp3*^LSL^)^7^. The *Foxp3*^*LSL*^ allele harbors a loxP site-flanked Thy1.1 reporter followed by a STOP cassette and a *Foxp3*^GFP^ reporter knocked into the endogenous *Foxp3* locus. In *Foxp3*^LSL^ mice, Thy1.1 reporter marks Treg “wannabe” cells with the transcriptionally active *Foxp3* locus yet lacking Foxp3 expression similar to the GFP^+^ cells in *Foxp3*^GFPKO^ mice. 4-hydroxytamoxifen (4-OHT) treatment of *Cd4*^creERT2^*Foxp3*^LSL^ mice led to the excision of the STOP cassette and punctual induction of the Foxp3 protein, converting Foxp3^−^ Thy1.1^+^ Treg “wannabe” cells into fully functional Foxp3 expressing GFP^+^ Treg cells (Figure 4e). Since X-linked *Foxp3* gene is subject to random X chromosome inactivation, healthy *Foxp3*^LSL/WT^ female heterozygote mice harbor both functional Foxp3-sufficient Treg cells and Treg ‘wannabe” cells expressing *Foxp3*^WT^ and *Foxp3*^LSL^ allele, respectively. Thus, we sought to investigate the temporal dynamics of the emerging Foxp3-dependent transcriptional features upon acquisition of Foxp3 expression by Treg “wannabe” cells in 4-OHT-treated female heterozygous *Cd4*^creERT2^*Foxp3*^LSL/WT^ mice. Resting and activated Foxp3^+^GFP^+^ cells and control Foxp3^−^GFP^−^Thy1.1^+^ cells with matching activation state were sorted from the *Cd4*^creERT2^*Foxp3*^LSL/WT^ and *Cd4*^WT^*Foxp3*^LSL/WT^ littermates, respectively, on days 3, 7, 14, and 28 post single 4-OHT administration and subjected to RNA-seq analysis (Figure 4f, Extended Data Figure 7c). The top Foxp3 degradation-sensitive “TIR1-down” and “TIR1-up” genes showed time-dependent increases and decreases in their expression in both resting and activated Foxp3^+^GFP^+^Thy1.1^−^ cells in comparison to time- and activation state-matched Foxp3^−^GFP^−^Thy1.1^+^ “wannabe” controls, respectively, consistent with a likely direct role for Foxp3 in regulating their expression (Figure 4g). Further analysis of the overall pace at which Foxp3 installation drives transcriptional changes in Treg “wannabe” cells toward a bona fide Treg profile revealed an unexpectedly prolonged timeline of approximately two weeks. While it remains formally plausible that Foxp3-dependent transcriptional programs are established more rapidly during normally differentiating Treg cells compared to Foxp3 induction in Treg “wannabe” cells, these observations suggest that the establishment of the Treg transcriptional program and functionality is critically dependent on Foxp3 during Treg cell maturation. In contrast, fully differentiated Treg cells in healthy adult mice may not rely on Foxp3 to the same extent. Moreover, the slow kinetics of the Foxp3-driven gene program acquisition highlights the necessity for direct Foxp3 targets to act in *trans* to regulate downstream, indirect Foxp3-dependent genes.

### A requirement for Foxp3 during Treg cell maturation

**Extended Data Figure 8.**
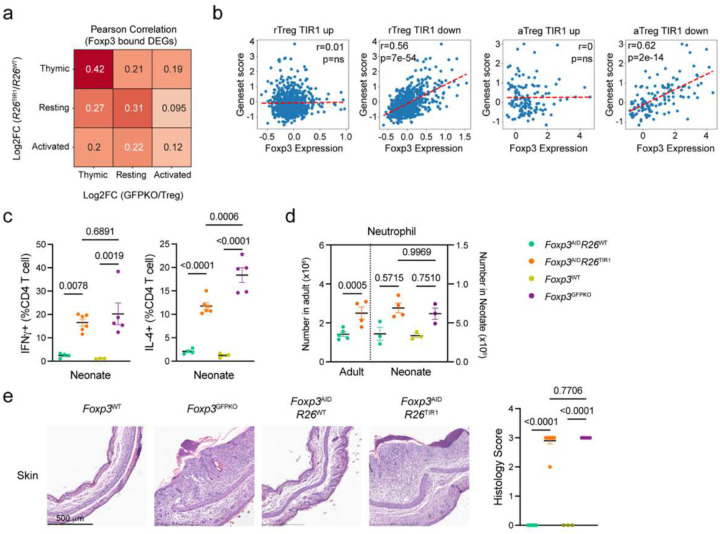
Foxp3 is preferentially required during Treg cell development. **(a)** Pearson correlation between Foxp3 degradation–induced and Foxp3-dependent DEGs in thymic, resting, and activated Treg cells, limited to Foxp3-bound genes. **(b)** Meta-cell analysis of resting and activated Treg scRNA-seq data from secondary lymphoid organs following Foxp3 degradation, correlating Foxp3 expression levels with “TIR1-up” and “TIR1-down” gene signatures identified in Figure 3. **(c)** Cytokine production by CD4^+^ T cells from neonatal *Foxp3*^AID^ mice after 14 days of Foxp3 degradation, in comparison to age-matched *Foxp3*^WT^ and *Foxp3*^GFPKO^ mice. **(d)** Neutrophil expansion in adult and neonatal *Foxp3*^AID^ mice following 14 days of Foxp3 degradation. Age-matched *Foxp3*^WT^ and *Foxp3*^GFPKO^ mice serve as controls for neonatal *Foxp3*^AID^ mice. **(e)** Representative H&E staining and histology scores of skin inflammation in neonatal *Foxp3*^AID^ mice after 14 days of Foxp3 degradation, in comparison to age-matched *Foxp3*^WT^ and *Foxp3*^GFPKO^ mice. (c–e) Scatter plots represent mean ± SEM; data are pooled from two independent experiments. One-way ANOVA.

**Figure 5.**
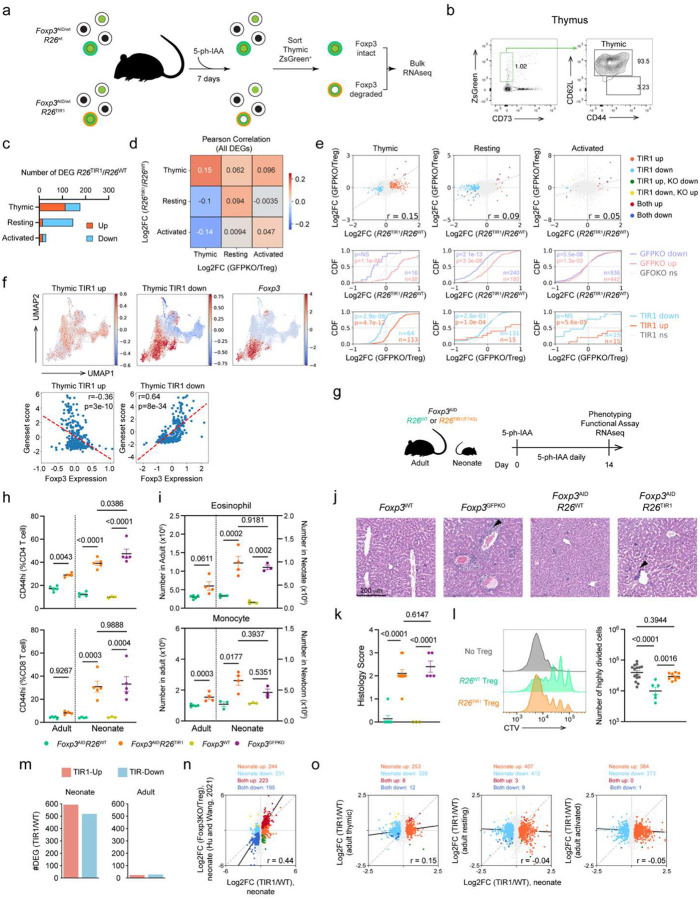
Foxp3 is preferentially required during early Treg cell development. **(a)** Experimental design for transcriptional profiling of developing thymic Treg cells. Each genotype consisted of three independent biological replicates. **(b)** Gating strategy used to sort CD73− nascent thymic Treg cells. (c) Bar graph comparing the number of Foxp3 degradation–induced DEGs in thymic, resting, and activated Treg cells. **(d)** Pearson correlation between Foxp3 degradation–induced and Foxp3-dependent DEGs in thymic, resting, and activated Treg cells. **(e)** Scatter and cumulative distribution function (CDF) plots comparing Foxp3 degradation–induced and Foxp3-dependent DEGs across the three Treg populations. **(f)** Meta-cell analysis of thymocyte scRNA-seq data^28^ correlating Foxp3 expression levels with the expression of TIR1-up and TIR1-down gene signatures identified in (a–c). UMAP plots are colored by expression levels of the TIR1-up, TIR1-down signatures, and Foxp3. **(g)** Experimental design for *in vivo* Foxp3 degradation in 1-day-old neonatal *Foxp3*^AID^ mice. **(h)** CD4 and CD8 T cell activation in adult and neonatal *Foxp3*^AID^ mice following Foxp3 degradation. **(i)** Expansion of eosinophils and neutrophils in adult and neonatal *Foxp3*^AID^ mice after Foxp3 degradation. **(j–k)** Representative H&E staining (j) and histology scores of liver inflammation (k) in neonatal *Foxp3*^AID^ mice following Foxp3 degradation. **(l)** *In vitro* suppression assay of Treg cells sorted from *Foxp3*^AID^*R26*^WT^ and *Foxp3*^AID^*R26*^TIR1(F74G)^ neonatal mice after 7 days of *in vivo* 5-ph-IAA administration. 5-ph-IAA was also included in culture to maintain Foxp3 degradation. (h–l) Data are pooled from two independent experiments. Scatter plots represent mean ± SEM. one-way ANOVA. **(m)** Bar graphs summarizing the number of Foxp3 degradation–induced DEGs in Treg cells from neonatal and adult *Foxp3*^AID^ mice. **(n)** Scatter plot correlating gene expression changes induced by Foxp3 degradation and Foxp3 gene deficiency^7^ in neonatal mice. **(o)** Scatter plots comparing gene expression changes induced by Foxp3 degradation in neonatal Treg cells to those in adult thymic, resting, and activated Treg cells.

To test whether Foxp3 is essential for the acquisition of Treg-specific transcriptional program and function during thymic Treg differentiation and peripheral maturation, we employed two complementary approaches to assess the effects of induced Foxp3 protein degradation in developing Treg cells. First, we investigated Foxp3 degradation-induced gene expression changes in developing Treg cells in the thymus. Following seven days of 5-ph-IAA mediated Foxp3 degradation in *Foxp3*^AID/WT^*R26*^TIR1(F74G)^ mice, we performed bulk RNA-seq analysis of developing ZsGreen^+^ CD73^low^ Treg cells isolated from the thymus (Figure 5a–b). Differential CD73 expression was used to discern recently generated nascent zsGreen^+^CD73^low^ CD62L^high^ Treg cells from recirculating CD73^high^ Treg cells entering the thymus from the periphery^27^. We then compared the expression of Foxp3 degradation-sensitive and Foxp3 dependent (*Foxp3*^GFPKO^ versus *Foxp3*^GFP^) genes across Treg developmental stages. The number of differentially expressed genes (DEGs) resulting from Foxp3 degradation was the highest in developing thymic Treg cells followed by resting Treg cells, whereas activated Treg cells exhibited the lowest number (Figure 5c). These data suggest that the transcriptional program of developing Treg cells is markedly more vulnerable to Foxp3 loss in comparison to mature Treg cells in the periphery, with the latter becoming even less dependent on continuous Foxp3 expression as they become activated. This observation was consistent with the absence of significant enrichment for Foxp3 binding in Foxp3 degradation-sensitive gene loci in activated Treg cells. Notably, Foxp3 degradation-sensitive genes and Foxp3-dependent genes exhibited the strongest correlation in developing thymic Treg cells, with this correlation progressively decreasing as Treg cells mature, reaching its lowest values in activated Treg cells (Figure 5d–e). This trend persisted even among Foxp3-bound genes (Extended Data Figure 8a). The declining correlation suggests that while Foxp3 deficiency closely mirrors the effects of Foxp3 degradation during early Treg differentiation, prolonged Foxp3 loss in mature or activated Treg cells may lead to secondary transcriptional changes beyond the primary Foxp3-regulated program. These findings underscore the importance of focusing on Foxp3 degradation-sensitive genes, rather than the broader set of Foxp3 dependent genes, to better capture its direct regulatory impact. Notably, Foxp3 dependent DEGs in developing Treg cells correlated poorly with Foxp3 degradation sensitive genes in resting and activated mature Treg cells, suggesting a switch of Foxp3 dependent regulatory mechanisms once Treg cells complete their differentiation and maturation. We then performed a meta-cell analysis to compare our data with previously published scRNA-seq datasets of thymic Foxp3^+^ Treg cells and their progenitors^28^. The “TIR1-up” (Foxp3 repressed) and “TIR1-down” (Foxp3 induced) Foxp3 degradation-sensitive gene signatures showed negative and positive correlation with Foxp3 expression, respectively, in a dose dependent manner suggesting a role for Foxp3 - both as a repressor and activator - in shaping the gene program of developing Treg cells (Figure 5f). Interestingly, similar meta-cell analysis of Foxp3-degradation induced transcriptional changes in resting and activated Treg cells revealed a correlation between Foxp3 dosage and the “TIR1-down” signature only (Extended Data Figure 8b). In contrast, the “TIR1-up” gene set showed no correlation with Foxp3 expression level. These findings further support a shift in a role of Foxp3 in gene regulation as Treg cells mature, particularly in its function as a transcriptional repressor.

Under physiologic conditions, the contribution of thymic output to the peripheral pool of Treg cells is minimal in adults. Instead, Treg cell maintenance is primarily afforded by the self-renewal of the existing differentiated Treg cells, whose transcriptional program and functionality were found resilient to the Foxp3 loss^7, 9, 29^ (Figures 1 and 2). In neonatal mice, Foxp3^+^ cells first appear among CD4SP thymocytes between day 2 and day 3 after birth; thymic Treg output continues to steadily increase reaching a plateau around day 21 with recently generated Treg cells accounting for the bulk of Treg peripheral pool^16^. Thus, we tested the requirement for Foxp3 expression in the suppressor function of early life Treg cells by treating neonatal *Foxp3*^AID^*R26*^TIR1(F74G)^ and *Foxp3*^AID^*R26*^WT^ control mice with 5-ph-IAA daily for two weeks, starting from day 1 after birth (Figure 5g). Contrary to adult mice, Foxp3 degradation in neonates led to severe autoimmune disease featuring pronounced T cell activation (Figure 5h, Extended Data Figure 8c), myeloproliferation (Figure 5i, Extended Data Figure 8d), and tissue inflammation (Figure 5j–k, Extended Data Figure 8e) similar to those in Foxp3-deficient *Foxp3*^GFPKO^ mice indicative of a loss of Treg function. The latter was confirmed by the lack of *in vitro* suppressor capacity of ZsGreen^+^ cells isolated from 5-ph-IAA treated *Foxp3*^AID^*R26*^TIR1(F74G)^ neonates (Figure 5l). Consistently, RNA-seq analysis of neonatal *Foxp3*^AID^*R26*^TIR1(F74G)^ and *Foxp3*^AID^*R26*^WT^ Treg cells subjected to seven days of *in vivo* Foxp3 degradation revealed hundreds of up- and down-regulated genes far exceeding the number of DEGs resulting from Foxp3 degradation induced in mature Treg cells in adult mice (Figure 5m). Foxp3 degradation-induced DEGs in early life Treg cells showed strong correlation with DEGs observed in Foxp3− Treg ‘wannabes’ from *Foxp3*^LSL^ neonates vs Foxp3^+^ Treg cells from *Foxp3*^DTR^ controls, confirming that the loss of Foxp3 in recently generated Treg cells phenocopies *Foxp3* genetic deficiency (Figure 5n). Among all maturation stages of adult Treg cells, Foxp3 degradation-induced DEGs in adult thymic Treg cells showed the highest concordance with those in neonates suggesting their similarity in terms of Foxp3 dependence of their transcriptional programs. In contrast, Foxp3 degradation-induced DEGs in adult resting and activated Treg cells showed no such similarity to the neonatal Foxp3-dependent gene expression features (Figure 5o). These results suggest that in early life, persistent Foxp3 expression is required for establishment of a stable gene regulatory network and functionality in recently generated Treg cells, likely by acting on its few direct targets and through continuous enforcement of initially unstable feed-forward regulation of indirect targets via intermediates acting in-*trans*^12^. Once this transcriptional network initially vulnerable to Foxp3 loss reaches a stable state in mature differentiated Treg cells, it is capable of preserving Treg functionality and Foxp3 dependent gene expression in the absence of Foxp3, except for a small group of direct Foxp3 targets.

### Foxp3 degradation induced tumor shrinkage with minimal adverse effects

**Figure 6.**
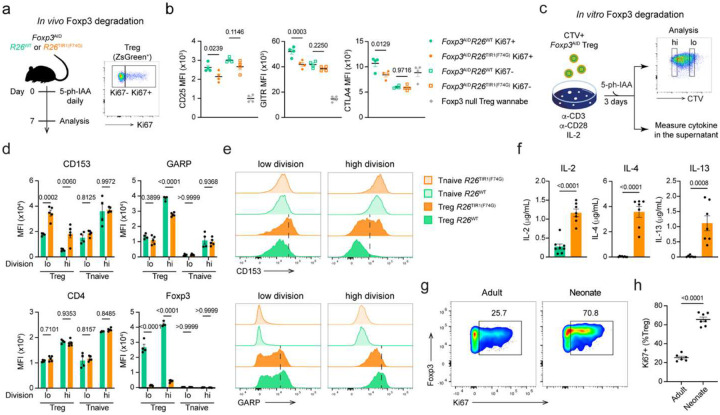
Dividing Treg cells are more reliant on Foxp3 for Treg specific gene expression. **(a)** Experimental design of proliferating Treg analysis *in vivo*. **(b)** Flow cytometry analysis of CD25, GITR, and CTLA4 protein levels in dividing versus non-deviding Treg cells following 7 days of *in vivo* Foxp3 degradation, in comparison to Foxp3-deficient Treg “wannabe” cells. Scatter plots represent mean ± SEM. Data are representative of two independent experiments. One-way ANOVA. **(c)** Experimental design of proliferating Treg cell analysis *in vitro*. **(d-e)** Combined data (d) and representative plots showing CD153, GARP, CD4, and Foxp3 protein levels in lowly and highly divided Treg cells. Similarly treated naïve CD4 T cells serve as Foxp3^−^ controls. Bargraphs represent mean ± SEM. Data are representative of two independent experiments. Two-way ANOVA. **(f)** IL-2, IL-4, and IL-13 concentrations in the supernatant of *in vitro* proliferating Treg assay. Bargraphs represent mean ± SEM. Data are pooled from two independent experiments. Two-tailed t test. **(g-h)** Representative plots (g) and combined data (h) showing Treg proliferation in 8-week-old adult versus 7-day-old neonatal mice measured by Ki67 positivity. Scatter plot represents mean ± SEM. Data are combined from two independent experiments. Two-tailed t test.

The observed dispensability of Foxp3 for the function of differentiated Treg cells in adult mice under physiological condition stood in a sharp contrast with our early finding of a loss of Treg cell function upon ablation of a conditional Foxp3 allele in differentiated Treg cells upon transduction with a Cre recombinase-expressing retroviral vector followed by an adoptive transfer into T cell-deficient recipients^14^. While Treg cells residing in lymphoreplete healthy mice undergo a slow turnover, they undergo pronounced proliferation in lymphopenic settings. Therefore, Foxp3 expression, while largely dispensable in relatively quiescent mature Treg cells, is likely needed to maintain developmentally established Treg specific transcriptional program in robustly dividing cells. To test this supposition, we first performed flow cytometric analysis of *Foxp3*^AID^ Treg cells following seven days of *in vivo* Foxp3 degradation, after parsing them into dividing and non-dividing cells on the basis of Ki67 expression (Figure 6a). While the overall phenotypic shift in Foxp3-degraded versus-replete Treg cells was modest, the proliferating Ki67^+^ subset accounted for most changes in CD25, GITR, CTLA4 protein levels, whereas the Ki67^−^ Treg subset underwent little change (Figure 6b), consistent with an idea that Foxp3 degradation affects primarily proliferating Treg cells. To corroborate these observations, we performed phenotypic analysis of cell trace violet (CTV)-labeled *Foxp3*^AID^ Treg cells stimulated to proliferate *in vitro* with anti-CD3 and anti-CD28 antibodies, alongside 5-ph-IAA treatment to induce Foxp3 degradation (Figure 6c). The highly divided (CTV^low^) cells showed a greater difference in Treg cell markers encoded by Foxp3 degradation-sensitive genes such as CD153 (*Tnfsf8*) and GARP (*Lrrc32*) compared to their lowly divided (CTV ^high^) counterparts (Figure 6d–e, Extended Data Figure 6d). Expression of CD4, serving as a control Foxp3-independent gene product, was unaffected by Foxp3 degradation regardless of the extent of Treg cell division (Figure 6d). Moreover, Foxp3-degraded Treg cells secreted more Foxp3-repressed proinflammatory cytokines including IL-2, IL-4, and IL-13 (Figure 6f, Extended Data Figure 6d). These data support the idea that Foxp3 is required for the maintenance of Treg identity and function preferentially during cell division. Of note, neonatal Treg cells were markedly more proliferative compared to adult Treg cells, evident by drastically higher percentages of Ki67^+^ cells (Figure 6g–h). Such enhanced proliferation together with other potential factors could contribute to their superior sensitivity to Foxp3 degradation.

**Extended Data Figure 9.**
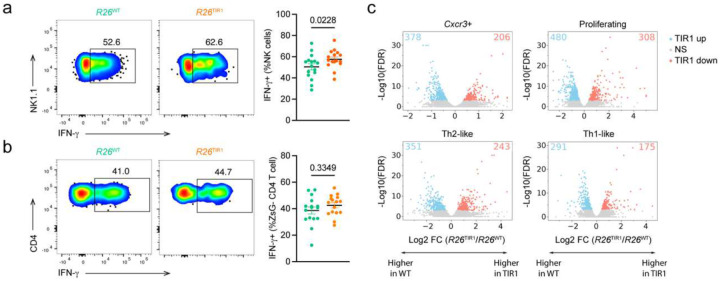
Foxp3 degradation leads to tumor shrinkage with minimal adverse effects. **(a)** Representative flow cytometry plots (left) and combined data (right) of IFN-γ production by tumor-infiltrating NK cells. Scatter plot shows mean ± SEM. Data are pooled from two independent experiments. Two-tailed *t*-test. **(b)** Representative flow cytometry plots (left) and quantification (right) of IFN-γ production by tumor-infiltrating ZsGreen^−^ CD4 T cells. Scatter plot shows mean ± SEM. Data are pooled from two independent experiments. Two-tailed *t*-test. **(c)** Volcano plts showing the number of genes up- and down-regulated in *Foxp3*^AID^*R26*^TIR1(F74G)^ tumor Treg cells within each clustered defined in Figure 7(l).

**Figure 7.**
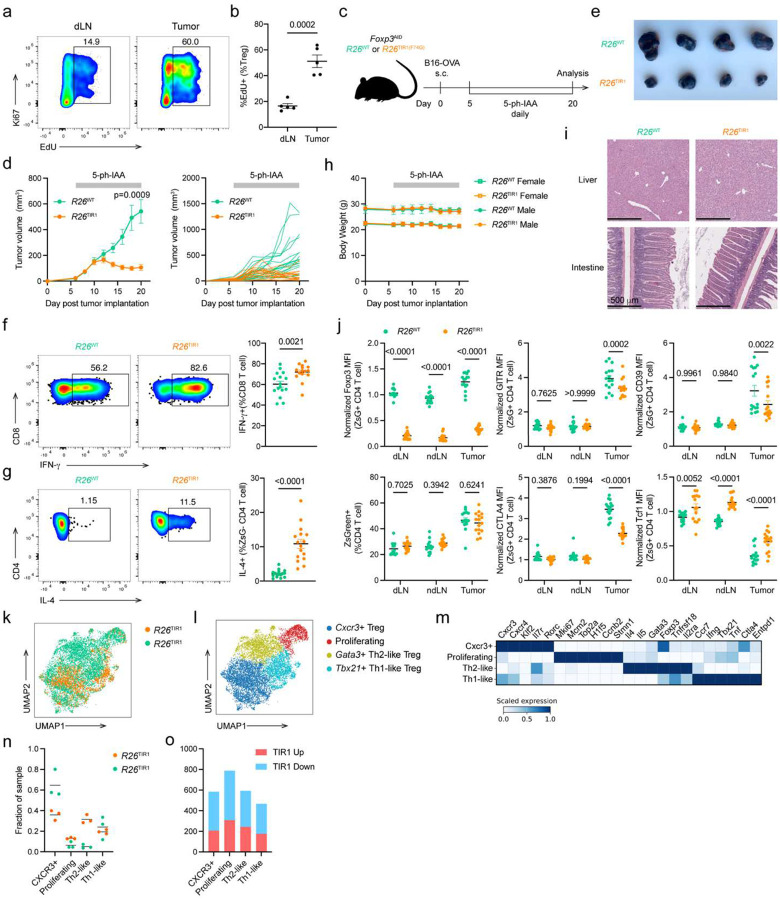
Foxp3 degradation leads to tumor shrinkage with minimal adverse effects. **(a–b)** Representative flow cytometry plots (a) and combined data (b) of Ki67 expression and EdU incorporation in Treg cells from the tumor and tumor-draining lymph node (dLN). Scatter plot shows mean ± SEM. Data are representative of two independent experiments. Two-tailed *t*-test. **(c)** Schematic of the tumor experiment design. **(d)** Tumor burden over time, shown as average (left) and individual (right) tumor growth curves. Line graph represents mean ± SEM. Data are pooled from two independent experiments. Two-way ANOVA (mixed-effects model) with Geisser-Greenhouse correction. **(e)** Representative tumor images on day 20. **(f)** Representative flow cytometry plots (left) and combined data (right) of IFN-γ production by tumor-infiltrating CD8 T cells. Scatter plot shows mean ± SEM. Data are pooled from two independent experiments. Two-tailed *t*-test. **(g)** Representative flow cytometry plots (left) and quantification (right) of IL-4 production by tumor-infiltrating ZsGreen− CD4 T cells. Scatter plot shows mean ± SEM. Data are pooled from two independent experiments. Two-tailed *t*-test. **(h)** Body weight monitoring throughout the experiment. Line graph shows mean ± SEM. Data are pooled from two independent experiments. **(i)** H&E staining of liver and intestine on day 20. **(j)** Expression levels of Foxp3, GITR, CD39, ZsGreen, CTLA-4, and Tcf1 in ZsGreen^+^ CD4 T cells from the dLN, non-draining lymph node (ndLN), and tumor on day 20. Scatter plots show mean ± SEM. Data are pooled from two independent experiments. Multiple *t*-tests. **(k–l)** UMAP visualization of scRNAseq analysis of tumor Treg cells from *Foxp3*^AID^*R26*^WT^ and *Foxp3*^AID^*R26*^TIR1(F74G)^ mice on day 14 after tumor implantation, colored by genotype (k) or cluster (l). **(m)** Heatmap showing gene expression profiles of each cluster in (l). **(n)** Proportional distribution of *Foxp3*^AID^*R26*^WT^ and *Foxp3*^AID^*R26*^TIR1(F74G)^ Treg cells within each cluster in (l). **(o)** Number of DEGs between *Foxp3*^AID^*R26*^WT^ and *Foxp3*^AID^*R26*^TIR1(F74G)^ Treg cells in each cluster shown in (l).

These results raised a question whether the resilient state of Treg cell transcriptional and functional program is lost in a disease state associated with a high rate of Treg cell turnover in adult mice. Solid organ tumors are highly enriched for activated Treg cells^30, 31^. Using both Ki67 staining and 5-ethynyl-2’-deoxyuridine (EdU) incorporation assay, we confirmed that tumoral Treg cells were markedly more proliferative in comparison to their counterparts residing in the tumor draining lymph node (dLN) (Figure 7a–b). Therefore, we reasoned that Foxp3 degradation may preferentially compromise the identity and function of tumoral Treg cells. To test this idea, we implanted B16-OVA melanoma cells in the flank of *Foxp3*^AID^*R26*^TIR1(F74G)^ and *Foxp3*^AID^*R26*^WT^ mice. On day 5 post tumor implantation, the tumor-bearing mice were treated daily with 5-ph-IAA to induce Foxp3 degradation (Figure 7c). While the tumors grow unabatedly in *Foxp3*^AID^*R26*^WT^ mice, tumors in *Foxp3*^AID^*R26*^TIR1(F74G)^ ceased to grow and underwent rapid shrinkage (Figure 7d–e). CD8 T cells and NK cells within the tumor exhibited heightened effector function, as evidenced by increased IFN-γ production (Figure 7f, Extended Data Figure 9a). Interestingly, ZsGreen− effector CD4 T cells, instead of upregulating IFN-γ, showed increased IL-4 expression (Figure 7g, Extended Data Figure 9b), which has been recently implicated in anti-tumor immunity^32, 33^. The enhanced T cell effector responses suggest that Foxp3 degradation boosts anti-tumor immunity by alleviating Treg-mediated immunosuppression. Importantly, severe adverse effects, typically seen upon pan-Treg cell ablation in cancer-bearing mice, were completely lacking as there were no signs of body weight loss, hunched posture, skin lesions, or tissue inflammation based on clinical or histological evaluations (Figure 7h–i). Although Foxp3 degradation had no effect on the abundance of ZsGreen^+^ Treg cells in the tumor, dLN and non-draining lymph nodes (ndLN), Foxp3 degradation induced phenotypic changes were markedly more pronounced in tumoral Treg cells. While increased TCF1 expression was observed in both non-tumoral and tumoral Treg cells, reduced CTLA4, GITR and CD39 expression was only observed in the latter (Figure 7j). As these molecules play critical roles in Treg-mediated immune suppression, these studies suggest that Foxp3 degradation boosts antitumor immunity with minimal immune related adverse effects by resulting in a selective loss of intratumoral Treg cell function.

To gain transcriptome-wide insights into the Foxp3-dependent gene program within intratumoral Treg cells, we performed scRNA-seq profiling of tumor-infiltrating Treg cells isolated from *Foxp3*^AID^*R26*^WT^ and *Foxp3*^AID^*R26*^TIR1(F74G)^ mice following 15 days of continuous Foxp3 degradation. In notable contrast to our observations in healthy adult mice — where Foxp3 degradation induced minimal transcriptional changes — loss of Foxp3 protein in tumor-bearing mice led to profound and widespread transcriptional alterations within tumor Treg cells visualized by UMAP (Figure 7k). To characterize the heterogeneity of tumor Treg cells with and without Foxp3 degradation, we performed a coarse clustering analysis, identifying four clusters: (1) a *Gata3*^hi^, Th2-like cluster; (2) a *Cxcr3*^hi^ cluster; (3) a *Tbx21*^hi^, Th1-like cluster; and (4) an *Mki67*^hi^, proliferating cluster (Figure 7l–m). Unlike steady-state Treg cells, where both Foxp3-sufficient and Foxp3-degraded cells remained similarly distributed across clusters even under refined clustering conditions, intratumoral Treg cells displayed a markedly uneven distribution depending on Foxp3 status, even at this coarse level of clustering. Specifically, the Th2-like (*Gata3*^hi^) cluster was overwhelmingly populated by Foxp3-degraded Treg cells from *Foxp3*^AID^*R26*^TIR1(F74G)^ mice, suggesting that Foxp3 loss preferentially skews tumor Treg cells toward a Th2-like state. Conversely, the *Cxcr3*^hi^ cluster was predominantly composed of Foxp3-sufficient Treg cells from *Foxp3*^AID^*R26*^WT^ mice. The proliferating (*Mki67*^hi^) cluster contained a slightly higher proportion of Foxp3-degraded Treg cells, while the Th1-like (*Tbx21*^hi^) cluster maintained a comparable representation of Treg cells from both genotypes (Figure 7n). Given developmental stage-dependent requirement for Foxp3 in maintaining Treg identity and functionality observed in healthy mice, we sought to determine whether Foxp3 exerts cell state context-specific roles across distinct tumoral Treg subsets. Thus, we performed pseudo-bulk differential gene expression analyses between *Foxp3*^AID^*R26*^WT^ and *Foxp3*^AID^*R26*^TIR1(F74G)^ tumor Treg cells within each cluster. Notably, the proliferating cluster revealed the highest number of DEGs (Figure 7o, Extended Data Figure 9c). These results confirm a heightened dependence on Foxp3 for the transcriptional maintenance of Treg cells undergoing active proliferation within the tumor microenvironment.

## Discussion

Previous genetic studies showed that Treg cell identity, suppressor function and fitness, indispensable for forestalling fatal autoimmune and inflammatory pathologies, are defined by the expression of their lineage specifying transcription factor (TF) Foxp3^1, 5, 34 4, 19^. Subsequent genetic fate mapping and temporally controlled installation of Foxp3 expression in Treg “wannabe” cells expressing *Foxp3*^LSL^ reporter null allele showed that Treg cells attain a stable differentiated cell state consistent with their indispensable function^7, 9^. Accordingly, Foxp3 protein expression has been associated with distinct transcriptional and chromatin features of Treg cells, yet a large number of Foxp3-depedent genes are not bound by Foxp3^4, 12^. Furthermore, Foxp3 binding across the genome, while sequence specific, is associated with transcriptional changes in only a few Foxp3-bound genes^12^. The latter have been proposed to propagate Foxp3-dependent genome-wide features in a “relay-like” manner as suggested by the finding of Foxp3-mediated repression of *Tcf7* gene accounting for half of all chromatin sites and genes repressed in Treg cells in a Foxp3-dependent manner^12^. Such transcriptional “staging” by Foxp3 would be expected to result in an unstable, readily reversible cell state especially in situations when Foxp3 expression is decreased or lost. Indeed, it has been suggested that in inflammatory or hypoxic settings mouse and human Treg cells can lose Foxp3 expression and become pro-inflammatory “ex-Treg” cells, which assume pathogenic potential and contribute to autoimmunity and inflammation^35, 36, 37^. On the other hand, it has been proposed that Foxp3 instability is limited to newly generated Treg cells and that after its transient loss re-expression of Foxp3 restores Treg suppressor function^8, 9^. The interpretation of these conflicting results has been confounded by limitations of differing genetic *Foxp3* ablation and fate mapping strategies employed in the respective studies and extended timescales of Foxp3 RNA and protein turnover obscuring separation of direct and indirect effects.

Our studies employing a novel inducible Foxp3 degradation model demonstrated that, contrary to a complete halt of functional Treg development resulting from germline or T cell-specific Foxp3 deficiency, Foxp3 is largely dispensable for the function and fitness of fully differentiated Treg cells in adult mice. This conclusion is supported by our findings that Foxp3 degradation induced upon 5-ph-IAA treatment of adult *Foxp3*^AID^*R26*^TIR1(F74G)^ mice for up to 4 weeks did not result in clinical manifestations of autoimmune disease or wasting, while *Foxp3*^*DTR*^ mice of similar age subjected to DT-induced Treg cell ablation succumbed to the disease within 2–3 weeks. Furthermore, suppressor capacity of adult Treg cells deprived of Foxp3 protein expression was unaffected when assessed on a per cell basis in an *in vitro* suppression assay. While immune activation in the adult mice was somewhat increased upon continuous Foxp3 degradation for 2 weeks, it was largely maintained without further exacerbation when Foxp3 degradation was extended to 4 weeks, likely reflecting an establishment of a new equilibrium between immune activation and suppression. Accordingly, Foxp3-dependent transcriptional features resulting from Foxp3 gene deficiency were barely affected by induced loss of Foxp3, which resulted in the changes of a small number of Treg signature transcripts, likely enriched for Foxp3 direct target gene products, and corresponding protein markers. Likewise, Treg cell function was unaffected by induced ablation of a conditional *Foxp3* allele in differentiated Treg cells in adult *Cd4*^*creERT2*^*Foxp3*^*fl*^ mice. This result suggests that after Foxp3 establishes the identity, fitness, and functionality of Treg cells during their differentiation, it becomes unessential, contrary to the widely accepted view of its continued necessity in fully differentiated Treg cells. In this regard, Foxp3 may belong to a category of transcription factors like PU.1 and Mash-1, whose critical role in initial commitment of B cell and neuronal cell lineages, respectively, becomes largely non-essential for the identity and core functions of their corresponding differentiated progenies^38, 39, 40, 41, 42, 43, 44, 45, 46, 47, 48^.

Notably, our studies revealed an important temporal exception to the notion of Foxp3 dispensability. Contrary to adult mice, analysis of peripheral Treg cells in *Foxp3*^AID^*R26*^TIR1(F74G)^ neonates demonstrated that the Foxp3-dependent transcriptional and functional programs are absolutely dependent on Foxp3, with its loss resulting in severe autoimmune inflammatory disease indistinguishable from that seen in mice with a congenital *Foxp3* deficiency. It seems unlikely that the observed disease can be accounted solely by the disruption of Foxp3 expression during thymic Treg generation, because the expression of TIR1 is controlled by a Foxp3-driven Cre. In addition, small number of functional Treg cells present in the periphery would be able to rescue the disease if they were resilient to Foxp3 loss. In this regard, adoptive transfer of small numbers of Treg cells into neonatal *Foxp3*^null^ mice or temporally induced installation of Foxp3 protein expression in a small cohort *Foxp3*^LSL^ expressing Treg “wannabe” cells affords protection from the disease^1, 7^. Furthermore, we found that Foxp3 protein degradation in both adult Foxp3-expressing thymocytes and early life peripheral Treg cells caused markedly more extensive transcriptional changes than those observed following Foxp3 degradation in fully differentiated adult peripheral Treg cells. Furthermore, Foxp3 protein degradation in early life Treg cells resulted in a profound loss of their functionality revealed by *in vitro* suppression assays. These findings suggest that stabilization of the Foxp3-dependent transcriptional program occurs at a relatively slow tempo. In support of this notion, it took approximately 2–3 weeks for the transcriptional program initiated by newly installed Foxp3 protein in Treg “wannabe” cells to approach that of bona fide Treg cells. Together, these results support a model whereby the initially vulnerable Foxp3-dependent gene regulatory network in Foxp3^+^ thymocytes and early life peripheral Treg cells gradually matures into a stable, largely Foxp3 independent state. This transition appears to unfold over an unexpectedly long timescale, possibly reflecting the “relay-like” propagation of Foxp3-mediated gene expression changes, whereby a small number of direct Foxp3 targets act in-*trans* to modulate broader networks of gene expression. Additionally, a relatively modest scale of modulation of a number of gene by Foxp3 can contribute to the slow tempo of the acquisition of Foxp3-dependent network resilience. Finally, early life peripheral T cells, almost exclusively made of recent thymic emigrants, produce limited amount of IL-2, which can further compromise maintenance of Treg transcriptional program upon removal of Foxp3^49^. In line with this, we found that this program encompasses genes, whose cis-regulatory elements are enriched for STAT binding motifs.

Maintenance of cell identity and function during cell division relies on faithful inheritance of transcriptional features in daughters through a wide range of epigenetic and genetic enforcement mechanisms^50^. In differentiated Treg cells, an intronic *Foxp3* enhancer CNS2 ensures heritable maintenance of Foxp3 expression in dividing cells. Thus, in addition to a likely extended time scale of establishment of Foxp3-dependent transcriptional program, higher proliferative activity detected in early life Treg population vs their adult counterparts could also contribute to the vulnerability of their Foxp3 dependent transcriptional program. Indeed, in differentiated adult Treg cells, the observed changes in expression of Treg cell markers were largely limited to dividing cells.

Among a wide range of biotic and abiotic challenges, activation of tumoral Treg cells prominently stands out based on gene expression and flow cytometry analyses^51, 52, 53^. Our flow cytometric analysis showed markedly enhanced proliferative activity of tumoral Treg cells in comparison to those residing in tumor-draining lymph nodes. The observed high rate of division of tumoral Treg cells was associated with their selective loss of function upon Foxp3 degradation manifested in tumor regression and enhanced anti-tumor immune responses in the absence of major adverse effects including inflammatory disease and wasting typically associated with wholesale ablation of Treg cells in experimental models of cancer^54, 55, 56, 57, 58, 59, 60, 61, 62, 63, 64^. Consistent with the impaired function of tumoral Treg cells subjected to Foxp3 protein degradation, we observed pronounced gene expression changes in tumor Treg cells in contrast to differentiated Treg cells in healthy mice. In addition to cell division, it is possible that other, yet to be determined factors present in the tumor microenvironment contribute to the vulnerability of Treg transcriptional and functional features to the loss of Foxp3 in the tumor microenvironment. Regardless of its mechanistic aspects, induced Foxp3 degradation offers a novel strategy for immunotherapy of tumors featuring highly activated and dividing Treg cells. This strategy affords therapeutic efficacy while minimizing adverse inflammatory and autoimmune sequelae.

In conclusion, our studies suggest that the initially vulnerable Foxp3 dependent gene regulatory network and associated functionality of Treg cells progress over time to a resilient state. Once this state is established, Treg cell function and much of the Foxp3-driven transcriptional program, except for few genes likely enriched for Foxp3 direct targets, are maintained even after the loss of Foxp3 expression. However, this resilience is lost in the tumor microenvironment associated with extensive Treg proliferation. These findings have important implications for understanding of Treg cell function in health and disease, including autoimmune and inflammatory disorder, and cancer.

## Methods

### Mice

All animal experiments in this study were approved by the Sloan Kettering Institute (SKI) Institutional Animal Care and Use Committee under protocol 08-10-023 or Yale University Institutional Animal Care and Use Committee under protocol 2023–20503. Mice were housed at the SKI or Yale University animal facility under specific pathogen free (SPF) conditions on a 12-hour light/dark cycle with free access to water and regular chow diet. *Foxp3*^DTR^, *Foxp3*^fl^, and *Cd4*^creERT2^ mice used in this study have been previously described^1, 19, 65^. *Foxp3*^AID^, *ROSA26*^TIR1^, and *ROSA26*^TIR1(F74G)^ mice were generated in this study. All control and experimental animals were age-matched, and littermates were used as controls unless otherwise indicated.

### Generation of *Foxp3*^AID^, *ROSA26*^TIR1^, and *ROSA26*^TIR1(F74G)^ mice

Gene targeting was carried out in 129/B6 F1 hybrid embryonic stem (ES) cells using a targeting vector spanning the Foxp3 locus. At the N-terminus, the auxin-inducible degron (AID) sequence was fused to Foxp3 via a seven–amino acid flexible linker (GSHGGSG). An IRES-ZsGreen-T2A-iCre-Frt-neo-Frt cassette was inserted into the 3′ UTR immediately downstream of the stop codon. ES clones with successful targeting were validated and injected into CD-1 tetraploid blastocysts to generate knock-in founders. These founders were then crossed with a Flp deleter line to excise the neo cassette. The resulting F1 progeny were backcrossed to the C57BL/6 background for at least three generations prior to *in vivo* experiments.

To generate the *ROSA26*^TIR1^ strain, a targeting construct was assembled by cloning a TIR1 (WT)-3xMyc-P2A-mCherry fragment into the FseI-linearized Ai32 targeting vector (Addgene #34880), positioned between a loxP-Stop-loxP cassette and a WPRE. The complete targeting vector included the *ROSA26* left homology arm, the CAG promoter, a loxP-Stop-loxP cassette, WPRE, a bovine growth hormone (bGH) polyadenylation signal, an AttB-neo-AttP drug selection cassette, and the *ROSA26* right homology arm. The linearized construct was transfected into albino C57BL/6 ES cells. Neomycin-resistant clones were screened by Southern blot, followed by PCR to confirm correct targeting. Karyotypically normal ES clones were used to generate chimeras, which were subsequently bred with albino C57BL/6 mice. Germline-transmitted founders were crossed with a PhiC31 deleter strain to remove the neomycin resistance cassette.

To generate the *ROSA26*^TIR1(F74G)^ strain, CRISPR-mediated gene editing was used to introduce a point mutation in ROSA26^TIR1/+^ zygotes, converting phenylalanine (F; **TTC**) to glycine (G; **GGC**) at position 74.

#### gRNA spacer sequence:

GAAGCGGCTGAAGTTGGAGC

#### HDR template sequence:

GCGGCGTCTTCGTGGGCAACTGCTACGCCGTGCGCGCCGGCCGCGTCGCCGCGCGGTT

CCCCAACGTGCGGGCGCTCACGGTGAAGGGGAAGCCACAC**GGC**GCCGACTTCAACCTCG

TGCCCCCCGACTGGGGCGGCTACGCGGGGCCGTGGATCGAGGCGGCCGCGAGGGGATG

CCACGGCCTGGAGGAGCTCAGGATG

In addition to the desired point mutation, a silent mutation (CCC to CCA) was included the HDR template to prevent re-cutting by Cas9. Offspring were screened by PCR amplification followed by KasI restriction digest. Confirmed mutants were validated by Sanger sequencing and bred to C57BL/6 mice to establish germline-transmitting founders.

### Treatment of mice with 5-phenyl-indoacetic acid (5-ph-IAA) and diphtheria toxin

Diphtheria toxin (DT, List Biological Laboratories, 150) was dissolved in PBS and administered intraperitoneally (i.p.) at a dose of 20 μg/kg on day 0. This was followed by six subsequent injections of 5 μg/kg every other day. Mice were euthanized for analysis on day 14.

5-Phenyl-indoleacetic acid (5-ph-IAA) (MedChemExpress, HY-134653) was dissolved with 0.2 M NaOH, diluted in PBS, and administered daily via i.p. injection at a dose of 10 mg/kg per mouse.

### Reagents and antibodies

The following antibodies and reagents were used in this study for flow cytometry, with clones, venders, catalog numbers and dilutions as indicated: anti-Siglec-F (E50-2440, BD, 562681, 1:400), anti-I-A/I-E (M5/114.15.2, Biosciences, 566086, 1:1,200), anti-NK1.1 (PK136, Thermo Fisher, 47-5941-82, 1:400), anti-CD45 (30-F11, BioLegend, 103136, 1:600), anti-CD11b (M1/70, BioLegend, 101257, 1:800), anti-CD11b (M1/70, BD Biosciences, 363-0112-82, 1:400), anti-CD3ε (17A2, BioLegend, 100237, 1:500), anti-γδTCR (GL3, BD Biosciences, 750410, 1:300), anti-Cd278 (C398.4A, BD Biosciences, 567918, 200), anti-TCR beta (H57–597, BD Biosciences, 748405, 1:300), Anti-TCR beta (H57–597, Thermo Fisher, 47-5961-82, 1:300), anti-TCR beta (H57–597, BioLegend, 109227, 1:200), anti-TCR beta (H57–597, Thermo Fisher, 12-5961-83, 1:400), anti-CD153 (RM153, BD Bioscience, 741575, 1:400), anti-CD24 (M1/69, Thermo Fisher, 46-0242-82, 1:800), anti-CD304 (3E12, BioLegend, 145209, 1:300), anti-CD3 (IM7, BioLegend, 103049, 1:400), anti-CD44 (IM7, BD Biosciences, 563971, 1:400), anti-CD44 (IM7, BioLegend, 103026, 1:100), anti-ZsGreen (polyclonal, Frontier Institute co.,ltd, MSFR106470, 1:800), anti-KLRG1 (2F1, Thermo Fisher, 35-5893-82), anti-CD39 (24DMS1, Thermo Fisher, 25-0391-82, 1:400), anti-TCF1 (C63D9, Cell signaling, 6709, 1:200), anti-IL-10 (JES5-16E3, BioLegend, 505021, 1:200), anti-CD4 (RM4-5, BD Biosciences, 414-0042-82, 1:400), anti-CD4 (RM4-5, BioLegend, 100536, 1:400), anti-CD4 (RM4-5, Thermo Fisher, 47-0042-82, 1:400), anti-CD4 (RM4-5, BioLegend, 100548, 1:400), anti-TNFa (MP6-XT22, BioLegend, 506329, 1:400), anti-IFNg (XMG1.2, BioLegend, 505836, 1:200), anti-IL-22 (1H8PWSR, Thermo Fisher, 46-7221-80, 1:400), anti-IL13 (eBio13A, Thermo Fisher, 12-7133-82, 1:400), anti-IL-4 (11B11, Thermo Fisher, 17-7041-82, 1:300), anti CD11c (N418, Thermo Fisher, 48-0114-82, 1:200), anti-Ly6c (HK1.4, BioLegend, 128037, 1:1,200), anti-Ly6C (HK1.4, BioLegend, 128041, 1:1000), anti-CD122 (TM-β1, BD Bioscience, 564763, 1:200), anti-GARP (YGIC86, Thermo Fisher, 25–9891, 1:200), anti-CD86 (GL1, Thermo Fisher, 12-0862-85, 1:400), anti-Ly6G (1A8, BioLegend, 127618, 1:500), anti-CD64 (X54–5/7.1, BioLegend, 139306, 1:200), anti-CD127 (A7R34, Tonbo Bioscience, 20–1271-U100, 1:200), anti-CD122 (5H4, Thermo Fisher, 13-1221-82, 1:200), anti-Guinea Pig (polyclonal, Thermo Fisher, SA5–10094, 1:1,000), anti-FR4 (12A5, BD Biosciences, 744121, 1:200), anti-FR4 (12A5, BD Biosciences, 560318, 1:200), anti-OX40 (OX-86, Thermo Fisher, 46-1341-82, 1:300), anti-CD120b (TR75-89, BD Bioscience, 564088, 1:200, anti-CD103 (M290, BD Biosciences, 566118, 1:300), anti-Ly-6C (HK1.4, BioLegend, 128037, 1:1,000), anti-CD90.2 (30-H12, BioLegend, 105320, 1:800), anti-CD90.2 (53–2.1, BD Biosciences, 564365, 1:1,500), anti-Foxp3 (FJK-16s, Thermo Fisher, 48-5773-82, 1:200), anti-Foxp3 (FJK-16s, Thermo Fisher, 17–5773-82, 1:200), anti-CD19 (6D5, BioLegend, 115510, 1:600), anti-F4|80 (BM8, BioLegend, 123133, 1:200), anti-CD4 (RM4–5, EBioscience, 564667, 1:400), anti-CD4 (RM4–5, BioLegend, 100553, 1:400), anti-CD8α (53–6.7, BioLegend, 100780, 1:600), anti-CD8α (53–6.7, BioLegend, 564297, 1:400), anti-CD8α (53–6.7, BioLegend, 100752, 1:500), anti-GITR (DTA-1, Thermo Fisher, 48-5874-82, 1:500), anti-CD73 (eBioTY/11.8, Thermo Fisher, 46-0731-82, 1:400), anti-CD73 (TY/11.8, BioLegend, 127208, 1:400), anti-CD62L (MEL-14, BioLegend, 104441, 1:100), anti-CD62L (MEL-14, BD Biosciences, 565213, 1:600), anti-CD62L (MEL-14, BD Biosciences, 741230, 1:800), anti-CD62L (MEL-14, BioLegend, 104441, 1:400), anti-CD62L (MEL-14, BioLegend, 104438, 1:1,600), anti-CTLA4 (UC10–4B9, BioLegend, 106323, 1:200), anti-CTLA4 (UC10–4B9, Thermo Fisher, 12-1522-82, 1:400), anti-Helios (22F6, BioLegend, 137216, 1:400), anti-Helios (22F6, BioLegend, 137236, 1:400), anti-Eos (ESB7C2, Thermo Fisher, 12-5758-82, 1:400), anti-Ki-67 (SolA15, Thermo Fisher, 61–5698, 1:2,000), anti Ki67 (B56, BD Biosciences, 563757, 1:1,000), anti-Ki67 (SolA15, Fisher Scientific, 15-5698-82, 1:8,000), anti-CD25 (PC61, BD Biosciences, 564022, 1:300; Thermo Fisher, 17-0251-82, 1:400), anti-PD-1 (29F.1A12, BioLegend, 135225, 1:400), anti-CD45 (30-F11, BioLegend, 103157, 1:1,000), anti-IL-2 (JES6–5H4, BioLegend, 503818, 1:400), streptavidin (Thermo Fisher, 46-4317-82, 1:1,000), Picolyl-Azide (Jena Bioscience, CLK-1288-5), CTV (Thermo Fisher, C34557), Zombie NIR dye (BioLegend, 423105, 1:1,000), Sytox Blue (Thermo Fisher, S34857), anti-mouse CD16/32 (2.4G2, Tonbo, 70-0161-M001, 1:500).

The following antibodies were used for ELISA capturing in this study: Purified anti-mouse IL-13 (14-7133-68, Invitrogen, 88-7137-88), Purified anti-mouse IL-4 (14-7041-68A, Invitrogen, 88-7044-88), Purified anti-mouse IL-2 (eBioscience, 14-7022-68). Purified anti-mouse IgE (R35–72, BD Pharmingen, 553413), goat anti-mouse IgG1 (2794408, Southern Biotech, 1070–01), Goat Anti-Mouse IgG3 (2794567, Southern Biotech, 1100–01), Goat anti-mouse IgG2a (2794475, Southern Biotech, 1080–01), goat anti-mouse IgG2b (2794517, Southern Biotech, 1090–01), Goat Anti-Mouse IgG2c (2794464, Southern Biotech, 1079–01), goat anti-mouse IgA (2314669, Southern Biotech, 1040–01), goat anti-mouse IgM (2794197, Southern Biotech, 1020–01).

The following antibodies were used for ELISA detection in this study: biotin anti-mouse IL-13 (13-7135-68A, Invitrogen, 88-7137-88), anti-mouse IL-4 (13-7042-68C, Invitrogen, 88-7044-88), anti-mouse IL-2 (eBioscience, 33-7021-68), Goat Anti-Mouse Ig (2728714, Southern Biotech, 1010–05), biotin rat anti-mouse IgE (R35–118, BD Pharmingen, 553419).

The following reagents were used to construct standard curves for ELISA in this study: mouse IL-4 lyophilized standard (39-8041-60, Invitrogen, 88-7044-88), mouse IL-13 lyophilized standard (39–7137/2EB-60, Invitrogen, 88-7137-88), mouse IL-2 (Thermo Fisher 212–12-5UG), purified Mouse IgG1, kappa, Isotype Control (15H6, Southern Biotech, 0102–01), purified mouse IgG2a, kappa, Isotype Control (UPC-10, Sigma, M5409), IgG2b Isotype Control from murine myeloma (MOPC-141, Sigma, M5534), Mouse IgG2c (6.3, AB_2794064, Southern Biotech, 0122–01), Purified Mouse IgG3, kappa, Isotype Control (A112–3, BD Pharmingen, 553486), Purified Mouse IgA, kappa, Isotype Control (M18–254, BD Pharmingen, 553476), IgM Isotype Control from murine myeloma (MOPC 104E, Sigma, M5909), Purified Mouse IgE, kappa, Isotype Control (C38–2, BD Pharmingen, 557079).

### Enzyme-linked immunosorbent assay (ELISA)

ELISA experiments for IL-2, IL-4 and IL-13 were performed in the following way. Cells were isolated from pooled secondary lymphoid organs (peripheral lymph nodes (cervical, axillary, brachial, inguinal), and spleen) and cultured on 96-well U bottom plate with 5% CO_2_ in a 200μl Cell Culture Media (complete RPMI 1640 Media supplied with 10% FBS, 100 U/mL penicillin–streptomycin, 2mM L-Glutamine, 10 mM HEPES buffer, 50 μM β-mercaptoethanol) and recombinant human IL-2 (0.5U/μl, Roche, C168121–01). Cells were treated with 5-ph-IAA (5 μM daily) in the presence of anti-CD3/CD28 activation beads (Thermo Fisher, 11452D). Culture was terminated after 3 days, and supernatant was used for the detection of forementioned cytokines. Briefly, 96-well flat bottom plate was coated with capture antibody in Coating Buffer (00-0000-53, Invitrogen, 88–7044-88) and incubated overnight at 4°C. Next day, plate was washed and blocked with ELISA/ELISPOT Diluent (00-4202-55, Invitrogen, 88–7044-88) at room temperature for 1 hour. Then, plate was washed, and serial dilutions of standards were performed by using of ELISA/ELISPOT Diluent. Next, samples were added to the plate and incubated overnight at 4°C. Next day, plate was washed, and detection antibody was added and incubated for 1 hour at room temperature. Next, plate was washed and incubated with streptavidin-HRP for IL-2 and IL-4 (00-50050-68, Invitrogen, 88-7044-88) or avidin-HRP for IL-13 (00-4100-94, Invitrogen, 88-7137-88) for 30 minutes at room temperature. Plate was then washed and incubated with TMB solution (00-4201-56, Invitrogen, 88-7044-88) at room temperature for 5–15 minutes. Finally, 1M H_3_PO_4_ (Sigma-Aldrich, P5811) was added to the plate to stop the colorimetric reaction.

Antibody ELISAs were conducted as previously described. Briefly, mouse peripheral blood was collected via cardiac puncture immediately after euthanasia into BD SST microcontainer tubes (02-675-185) and sera were harvested after centrifugation. Flat-bottom 96-well plates were coated with capturing antibodies in 50 μL 0.1 M NaHCO3 solution at pH 9.5 O/N at 4°C. The plates were then emptied, blocked with 200 μL 1% bovine serum albumin (VWR, 97061–422) in PBS, and washed 3 times with PBS containing 0.05% Tween-20 (Sigma-Aldrich, P1379). 50 μL of sera at appropriate dilutions was added and incubated O/N at 4°C. The plate was then incubated with 50 μL of biotinylated detection antibodies at 37°C for 2–3 hours, followed by 50 μL of avidin-HRP (Thermo Fisher, 18-4100-51) at 37°C for 30 minutes, and 100 μL of TMB solution (Thermo Fisher, 00-4201-56) at room temperature, with 3–4 washes with PBS-Tween in between each incubation steps. The colorimetric reaction was stopped with 100 μL of 1M H_3_PO_4_ after 5–10 minutes.

Absorbance at 450 nm was measured with a Synergy HTX plate reader (BioTek). Concentrations of antigens were determined using standard curves constructed with purified recombinant proteins and calculated with Gen5 3.02.2 (BioTek).

### Isolation of cells from lymphoid organs, lungs, and tumors

For flow cytometry analyses, animals were euthanized and perfused with 20 ml PBS. Cells were isolated form lymphoid organs by meshing with syringe plunger through 100 μm cell strainer (Corning, 07-201-432). Lungs and tumors were digested in RPMI 1640 with 2% FBS,10 mM HEPES buffer, 100 U/mL penicillin-streptomycin, 2 mM L-glutamate, 0.2 U/mL collagenase A (Sigma, 11088793001) and 1U/mL DNase I (Sigma-Aldrich, 10104159001) for 45 min at 37°C with vigorous shaking at 250 r.p.m. 6.35mm ceramic beads (MP Biomedicals, 116540034) were included to help with tissue dissociation. The digested lungs were filtered through 70 μm separation filters (Miltenyi Biotec, 130-095-823), washed and centrifuged in PBS-adjusted 40% Percoll (Sigma Aldrich, 17-0891-01) to enrich for lymphocytes. Erythrocytes from spleen, lung and liver were lysed by using of ACK lysis buffer (150 mM NH_4_Cl (Sigma-Aldrich, A9434), 10 mM KHCO_3_ (Sigma-Aldrich, P7682) and 0.1 mM Na_2_EDTA at pH 7.4).

For flow cytometry analysis, cells were stained with Zombie NIR dye in PBS for 10 min at 4°C to identify the dead cells followed by staining with anti-mouse CD16/32 in Staining Buffer (PBS with 0.2% BSA, 10 mM HEPES buffer and 2 mM EDTA) for 10 min at 4°C to block the Fc receptors. Next, cells were stained with fluorescently conjugated antibodies detecting cell surface antigens for 30 minutes at 4°C. To access the intracellular antigens, cells were fixed and permeabilized with eBioscience transcription factor staining buffer set (00-5523-00) according to the manufacturer’s instructions. Samples were recorded on Aurora cytometer (Cytek) by using of SpectroFlo software v3.1.2 and analyzed in FlowJo V10.10.0.

For cell sorting, cells isolated from pooled peripheral lymph nodes (cervical, axillary, brachial, inguinal) and spleen were enriched for CD4^+^ T cells using a mouse CD4+ T Cell Isolation Kit (Miltenyi, 130-104-454) according to the manufacturer’s instructions. Next, samples were stained with antibodies in Staining, washed and resuspended in a Sytox Blue containing (1:8,000) staining buffer to exclude dead cells. T_reg_ cells (CD4^+^TCRβ^+^ZsGreen^+^ from *Foxp3*^AID^*ROSA26*^WT^ mice and CD4^+^TCRβ^+^ZsGreen^+^mCherry^+^ from *Foxp3*^AID^*ROSA26*^TIR1(F74G)^ mice) and naïve CD4^+^ T cells (CD4^+^TCRβ^+^ZsGreen^−^CD44^lo^CD62L^hi^) were sorted into cell culture media.

### Flow cytometric analysis of cytokine production

To measure cytokine production following ex-vivo stimulation, single cell suspension was incubated with 5% CO_2_ at 37°C for 4 hours in the Cell Culture Media (200μl per well) supplied with 50ng/ml phorbol-12-myristate-13-acetate (Sigma-Aldrich, P8139), 500ng/ml ionomycin (Sigma-Aldrich, I0634), 2μM monensin (Sigma-Aldrich, M5273), 1μg/ml brefeldin A (Sigma-Aldrich, B6542). Cells were stained for flow cytometry as described above except for the fixation/permeabilization step and cytokine staining in which case BD Cytofix/Cytoperm Kit (BD Biosciences, 554715) was used according to the manufacturer’s instructions.

### *Ex vivo* CTV labeling experiment to track Treg cell proliferation

Sorted Treg cells were labeled with CTV (5 μM) and cultured with 5% CO_2_ on 48-well flat bottom plate pre-coated with anti-CD3/CD28 antibody with concentration of 5μg/ml each of anti-CD3 (145–2C11, BioXcell, BE0001–1) and anti-CD28 (37.51, BioXcell, BE0015–1). Culture was maintained in Cell Culture Media (600 μl per well) supplied with a recombinant human IL-2 (0.5U/μL). Cells were treated with 5-ph-IAA daily (5 μM). The first part of the culture was terminated after 16 hours to capture undivided cells and the cells which divided once. Another part of the culture was terminated after 72 hours to capture the cells dividing twice and more. Cells were stained for analysis as described above.

### *In vitro* suppression assay

Cells were isolated from pooled secondary lymphoid organs and enriched for CD4^+^ T cells as described above. Resting Treg cells (CD4^+^TCRβ^+^ZsGreen^+^CD62L^hi^CD44^lo^) were FACS sorted and 40,000 cells per well were plated on 96-well U bottom plate. 40,000 sorted naïve CD4 T cells from CD45.1 *Foxp3*^DTR^ mice (CD4^+^TCRβ^+^GFP^−^CD62L^hi^CD44^lo^) and 100,000 red blood cell-lysed splenocytes from *Tcrb*^−/−^*Tcrd*^−/−^ mice were added to the culture. Next, anti-CD3 (145–2C11, BioXcell, BE0001–1) antibody was added to each well to a final concentration of 1μg/ml. Cells were treated with 5-ph-IAA daily (5 μM). Cells were incubated in Cell Culture Media (200 μl per well) for 72 hours with 5% CO_2_ and then prepared for flow cytometry as described above. CTV-labeled Naïve CD4^+^ T cells which were divided more than 4 times were used to calculate T_reg_ cell mediated suppression with the following formula:

$$\%\text{Suppression}(\text{Sample}\;X)=\frac{\%\text{divided}(\text{no}\;\text{Treg})-\%\text{divided}(\text{Sample}\;X)}{\%\text{divided}(\text{no}\;\text{Treg})}$$

### Histology

Tissues were fixed in 10% neutral buffered formalin, transferred into 70% alcohol and sent to the HistoWiz for the downstream services. Briefly, tissues were embedded in paraffin and sectioned into 5μm slices followed by hematoxylin and eosin staining. Lymphocytic infiltration was blindly scored with the following criteria: 0, normal; 1, mild increase; 2, moderate increase; 3, severe increase.

### 5-Ethynyl-2’-deoxyuridine (EdU) labeling

Mice were injected i.p with EdU (5mg/mouse, MedChemExpress, HY-118411) for three consecutive days. Cells were stained with surface and intracellular antibodies as described above. To stain for EdU, click reaction was performed after staining with intracellular antibodies. Briefly, cells were incubated in the Click Reaction Buffer (CuSO_4_ 16.67mM (Fisher Science Education, 7758-98-7), BTTAA (2-(4-((bis((1-(tert-butyl)-1H-1,2,3-triazol-4-yl)methyl)amino)methyl)-1H-1,2,3-triazol-1-yl)acetic acid) 41.67mM (Jena Bioscience, CLK-067–100), picolyl-azide alexa-fluor 555 1.25 μM (Jena Bioscience, CLK-1288–5), sodium ascorbate 100mM (Spectrum, 134-03-2), H_2_O, PBS PH7.4) for 1 hour at room temperature followed by extensive washing before recording data on the cytometer.

### B16 melanoma model

B16-OVA melanoma cells were cultured in RPMI Medium supplemented with 10% fetal bovine serum (FBS; Gibco), 100U/mL penicillin-streptomycin at 37°C in a humidified incubator with 5% CO_2_. Cells were maintained in logarithmic growth phase and harvested at ~70–80% confluency using 0.05% trypsin-EDTA. Cells were washed twice with sterile phosphate-buffered saline (PBS) and resuspended in PBS 5 × 10^6^ cells/mL. For tumor implantation, 100 μL of the cell suspension (5 × 10^5^ cells) was injected subcutaneously into the right flank of mice using a 27G needle. Starting from day 5 post tumor implantation, mice were injected daily with 5-ph-IAA.Mice were monitored daily for general health and tumor growth. Tumor dimensions were measured using calipers, and volume was calculated using the formula: (length × width^2^) / 2.

### Bulk RNA sequencing and data analysis

For bulk RNA-sequencing from adult female *Foxp3*^AID/+^*ROSA26*^WT^ and *Foxp3*^AID/+^*ROSA26*^TIR1(F74G)^ mice, single cell suspensions from pooled secondary lymphoid organs or thymus were enriched for CD4^+^ T cells as described above. CD4^+^TCRβ^+^ZsGreen^+^CD62L^hi^CD44^lo^ resting Treg cells and CD4^+^TCRβ^+^ZsGreen^+^CD62L^lo^CD44^hi^ activated Treg cells from the secondary lymphoid organs, as well as CD4^+^TCRβ^+^ZsGreen+CD73^−^ nascent Treg cells from the thymus, were double sorted into TRIzol Reagent (Thermo Fisher, 15596–018). RNA extraction was then performed according to the manufacturer’s instructions. Briefly, phase separation in cells lysed in 1mL TRIzol Reagent was induced with 200 μL chloroform. RNA was extracted from 350 μL of the aqueous phase using the miRNeasy Micro Kit (Qiagen catalog # 217084) on the QIAcube Connect (Qiagen) according to the manufacturer’s protocol. Samples were eluted in 15–18 μL RNase-free water.

After RiboGreen quantification and quality control by Agilent BioAnalyzer, 1–2 ng total RNA with RNA integrity numbers ranging from 5.5 to 9.3 underwent amplification using the SMART-Seq v4 Ultra Low Input RNA Kit (Clonetech catalog # 63488), with 12 cycles of amplification. Subsequently, 4.5–8 ng of amplified cDNA was used to prepare libraries with the KAPA Hyper Prep Kit (Roche 07962363001) using 8 cycles of PCR. Samples were barcoded and run on a NovaSeq 6000 in a PE100 run, using the NovaSeq 6000 S4 Reagent Kit (200 cycles) (Illumina). An average of 58 million paired reads were generated per sample and the percent of mRNA bases per sample ranged from 79% to 87% and ribosomal reads averaged 0.35%.

### Single cell RNA sequencing, ATAC-sequencing and data analysis for steady state Treg cells

*Foxp3*^AID/+^*ROSA26*^WT^ and *Foxp3*^AID/+^*ROSA26*^TIR1(F74G)^ mice were treated with 5-ph-IAA for 0, 3, and 7 days. Enriched CD4 T cells from the spleen and lymph nodes were barcoded using Hash Tag Oligonucleotides (Biolegend). FACS-sorted *Foxp3*^AID^*R26*^WT^ and *Foxp3*^AID^*R26*^TIR1(F74G)^ Treg cells (four biological replicates of each genotype) within each time point were multiplexed together, washed once with PBS containing 0.04% bovine serum albumin (BSA), and resuspended in PBS containing 0.04% BSA to a final concentration of 700–1,200 cells/μl. For each time point, RNA sequencing was performed in one lane on Chromium instrument (10X genomics) following the user guide manual for 3′ v3.1. The viability of cells was above 80%, as confirmed with 0.2% (w/v) Trypan Blue staining (Countess II). Following reverse transcription and cell barcoding in droplets, emulsions were broken and cDNA purified using Dynabeads MyOne SILANE followed by PCR amplification per manual instruction.

Approximately 30,000 cells were targeted. Final libraries were sequenced on Illumina NovaSeq 6000 - S4 platform (R1 – 28 cycles, i7 – 8 cycles, R2 – 90 cycles). Viable cells were identified on the basis of library size and complexity, whereas cells with >20% of transcripts derived from mitochondria were excluded from further analysis.

For sample demultiplexing, HTO sequencing data were aligned to the HTO barcodes, and UMIs were counted for each cell using CITE-seq-Count. Using a two-component K-means algorithm, we partitioned logged HTO counts into two distributions: background noise (lower mean) and positive tags (larger mean). Each droplet was then assigned to its source sample based on tags in the positive signal component. We classified droplets with multiple assignments as doublets and those with a single assignment as singlets. This analysis was performed using SHARP v0.1.1.

Single-cell ATAC and gene expression profiling were performed using the 10X Genomics Next GEM Single Cell Multiome platform, following the Kits User Guide. Briefly, single cells suspension (viability >80%) were lysed for 4min and resuspended in Diluted Nuclei Buffer (10x Genomics, PN-2000207). Lysis efficiency and nuclei concentration was evaluated on Countess II automatic cell counter by trypan blue staining. Nuclei were loaded into transposition reaction, targeting recovery of 10,000 nuclei after encapsulation. After transposition reaction nuclei were encapsulated and barcoded. Next-generation sequencing libraries were constructed following User Guide, which were sequenced on an Illumina NovaSeq 6000 - S4 platform.

### Single cell RNA sequencing and data analysis for intratumoral Treg cells

#### Library preparation and sequencing

*Foxp3*^AID/y^*R26*^TIR1(F74G)^ and *Foxp3*^AID/y^*R26*^WT^ mice (3 per group) were injected subcutaneously with 1.5 × 10^6^ B16-OVA cells in 100 μL sterile PBS. Starting on day 5 post-implantation, 200 μg 5-ph-IAA in 200 μL was injected intraperitoneally daily. On day 14 post-implantation, the tumors were processed and prepared for sequencing using the GEM-X Flex assay (10X genomics). Following dissociation into single cell suspensions, cells were stained with fluorescent and TotalSeq-C anti-mouse hashtag antibodies (BioLegend) and washed extensively. Cells from each mouse were stained with a unique hashtag antibody. Cells were pooled and fixed in Fixation Buffer B (10X genomics) at 4C for 18 hours. After quenching and washing of cells per manufacturer instructions (10X genomics, Demonstrated Protocol CG000782 Rev A), cells were resuspended in 500 μL Quenching Buffer B. Reporter positive live/dead stain negative Tregs (ZombieNIR^−^CD45^+^CD3^+^TCRβ^+^CD8α^−^CD4^+^ZsGreen^+^ mCherry^+^ for *R26*^TIR1(F74G)^ mice or mCherry^−^ for *R26*^WT^ mice) were isolated from fixed single cell suspensions using FACS and sorted into PBS with 1% nuclease-free BSA Fraction V (Millipore Sigma, 126609) and 0.2 U/μL RNase-inhibitor (Millipore Sigma, PN-3335399001). Following FACS isolation, cells were spun down, resuspended in 200 μL of Quenching Buffer B, and pooled to normalize cell numbers between different mice. The remaining steps were performed per manufacturer instructions (10X genomics, User Guide CG000788 Rev A). In brief, cells were hybridized for 18 hours, washed, and encapsulated in one lane targeting 60,000 cells. Indexing PCR steps were performed using 9 amplification cycles for both the gene expression and cell surface protein libraries. Final sequencing libraries were sequenced on an Illumina NovaSeq X Plus System (index 1: 10 cycles, index 2: 10 cycles, read 1: 28 cycles, read 2: 88 cycles). Mean reads per cell for the gene expression and cell surface protein libraries were 32,505 (65.8% sequencing saturation) and 6,679 (18.6% sequencing saturation) respectively.

#### Data preprocessing

FASTQ files were processed using Cell Ranger v9.0 and reads were aligned to the Chromium Mouse Transcriptome Probe Set v1.1.0 GRCm39–2024-A (10X Genomics). Cells containing fewer than 1000 UMI counts or 1000 unique genes were filtered out. Cells containing more than 5% mitochondria-derived transcripts were filtered out. Genes that were expressed in more than 5 cells were retained for further analysis. Hashtag antibody data was demultiplexed using HashSolo with the following prior probabilities (negative: 0.01, singlet: 0.81, doublet: 0.18). Cells called as doublets or negative were filtered out. The resulting counts matrix consisted of 35,367 cells × 19,088 genes and was normalized to median UMI counts. The normalized data were then ln(1 + counts) transformed for downstream analysis.

### Statistics

Statistical significance was determined using tests indicated in the respective figure legends. P-values for *t*-test and ANOVA were calculated with GraphPad Prism 7 and had been corrected for multiple hypothesis testing.

## Extended Data
